# Inflammatory and Proliferative Pathway Activation in Human Esophageal Myofibroblasts Treated with Acidic Bile Salts

**DOI:** 10.3390/ijms231810371

**Published:** 2022-09-08

**Authors:** Madhura Patankar, Meng Li, Atousa Khalatbari, Joshua D. Castle, Liping Hu, Chunying Zhang, Anisa Shaker

**Affiliations:** 1 Department of Internal Medicine, Division of Gastrointestinal and Liver Diseases, Keck School of Medicine of USC, Los Angeles, CA 90033, USA; 2 USC Libraries Bioinformatics Services, University of Southern California, Los Angeles, CA 90007, USA

**Keywords:** GERD, esophagus, myofibroblast, inflammation

## Abstract

Subepithelial human esophageal myofibroblasts (HEMFs) in gastroesophageal reflux disease (GERD) are exposed to luminal contents via impaired squamous epithelium barrier integrity. The supernatant of HEMFs treated with acidic bile salts reflective of in vivo reflux increases squamous epithelial thickness. We aimed to identify the involved mechanisms using an unbiased approach. Acidic-bile-salt-treated primary HEMF cultures (n = 4) were submitted for RNA-Seq and analyzed with Partek Flow followed by Ingenuity Pathway Analysis (IPA). A total of 1165 molecules (579 downregulated, 586 upregulated) were differentially expressed, with most top regulated molecules either extracellular or in the plasma membrane. Increases in HEMF CXCL-8, IL-6, AREG, and EREG mRNA, and protein secretion were confirmed. Top identified canonical pathways were agranulocyte and granulocyte adhesion and diapedesis, PI3K/AKT signaling, CCR5 signaling in macrophages, and the STAT3 pathway. Top diseases and biological functions were cellular growth and development, hematopoiesis, immune cell trafficking, and cell-mediated response. The targets of the top upstream regulator ErbB2 included CXCL-8, IL-6, and AREG and the inhibition of CXCL-8 in the HEMF supernatant decreased squamous epithelial proliferation. Our work shows an inflammatory/immune cell and proliferative pathways activation in HEMFs in the GERD environment and identifies CXCL-8 as a HEMF-derived chemokine with paracrine proliferative effects on squamous epithelium.

## 1. Introduction

Gastroesophageal reflux disease (GERD) has a prevalence of up to 33% worldwide [1], with presentations ranging from nonerosive or endoscopy-negative reflux disease [2], to endoscopically visible complications of erosive esophagitis, peptic stricture, and Barrett’s esophagus [3]. Bleeding erosive esophagitis or Barrett’s esophagus can occur in up to 15.5% [4,5] and 5.6% [6,7] of cases, respectively, depending on the population investigated. Injurious agents invoked in the pathogenesis of mucosal damage encountered in subtypes of GERD include acid and bile salts [8] as well as other proposed factors, such as pepsin and the microbiome, which remain less well-defined and studied [9,10].

The pathogenesis of mucosal injury in GERD is complex [2,11]. Animal [8] and human histopathologic studies [12,13] support the concept that esophageal damage in GERD is not simply a chemical injury but rather a cytokine-mediated response, whereby damage is inflicted by inflammatory cells that have infiltrated the mucosa in response to cytokines produced by squamous epithelial cells exposed to acid and bile reflux. The mechanisms by which squamous epithelial cells respond to refluxate has traditionally not accounted for the role of the underlying, poorly characterized subepithelial cells. Squamous epithelium in both nonerosive and erosive GERD is characterized by an increase in thickness and basal layer hyperplasia [14]. The barrier integrity and function of GERD epithelium is impaired, however, and presents histologically as dilated intercellular spaces (DIS) [15] and functionally with an increase in permeability reflected by a reduced mucosal impedance [16] and increased paracellular flux of molecules [17]. This impaired barrier integrity also allows for the penetration of noxious luminal agents into the deeper basal layers of the squamous epithelium [18] and the underlying subepithelial space [19]. Subepithelial cells can in turn influence overlying epithelial cells and surrounding nonimmune and immune stromal cells.

We have previously shown that a subtype of subepithelial cells, human esophageal myofibroblasts (HEMFs), are increased in biopsies from patients with histologic features of GERD. These spindle-shaped stromal cells are defined and distinguished from fibroblasts and other stromal cells, respectively, by characteristic intracellular protein (α-SMA+, vimentin+, desmin−) and cell surface marker (CD90+, CD45−, CD31−) expression patterns [19,20], with a pattern of expression similar to that of subepithelial murine stromal cells [21]. HEMFs treated with acidified media increase the secretion of a number of proinflammatory mediators including CXCL-8 (IL-8) and IL-6. The relevance of this population of cells has been further highlighted by in vitro studies utilizing 3D-organotypic models which reproduce human stratified squamous epithelium. Untreated HEMFs support squamous epithelial growth in these models via paracrine mechanisms mediated at least in part via the secretion of the bone morphogenetic protein inhibitor, GREMLIN1 [22]. More recently, the effect of HEMF secreted factors on squamous epithelium was investigated in a modified 3D organotypic model of GERD. HEMFs treated with noxious stimuli such as acidic bile salts encountered in GERD secreted factors that increased the total squamous epithelial thickness, with an expansion of both basal and suprabasal layers [23]. An increase in total epithelial thickness has been reported as a marker of both nonerosive and erosive disease [14].

To begin to decipher the contribution of HEMFs to the mucosal response in GERD, we aimed to determine the signaling pathways activated in HEMFs in response to acidic bile salts. We used previously established primary HEMF cultures to identify these pathways via an unbiased approach with RNA sequencing followed by a validation of the identified genes in these cultures as well as at the mRNA and protein level in a previously described and validated immortalized HEMF cell line [19,20,22,23,24]. Our goal was to identify differentially expressed genes and molecular pathways in HEMFs activated in response to acidic bile salts, a major component of reflux, and thus better understand the role of HEMFs in the mucosal response in GERD. Our work shows an inflammatory/immune cell and proliferative pathways activation in HEMFs in the GERD environment and identifies HEMF-derived factors with direct paracrine effects on the proliferation of esophageal squamous epithelial cells.

## 2. Results

### 2.1. Differential Expression of Genes in Untreated and Acidic-Bile-Salt-Treated HEMF

**Figure 1:**
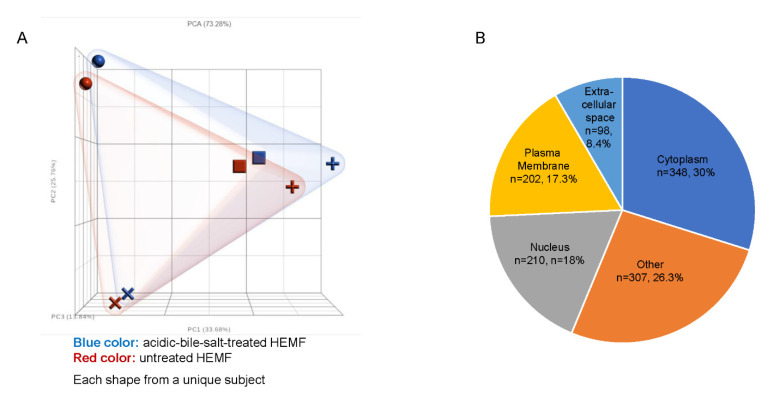
RNA sequencing of HEMFs. (**A**) Principal component analysis (PCA) plot of untreated (red) and treated (blue) primary HEMFs. Each shape (circle, square, cross, and plus sign) represents HEMFs isolated from a different human esophagus, with variability between samples shown by principal component percentage (PC%). (**B**) Location of differentially expressed genes.

Primary human esophageal myofibroblast (HEMF) cultures (n = 4) treated with acidic bile salts were submitted for RNA sequencing. A principal component analysis (PCA) scatter plot shows the expected variability between primary cultures established from unique esophagi (HEMFs 1–4), with a clear separation between acidic-bile-salt-treated and untreated samples for each primary culture (Figure 1A). An ANOVA identified a total of 1334 differentially expressed genes (DEGs, 664 upregulated and 670 downregulated) using fold changes of >1.2 and <−1.2 and *p* value < 0.05. After inputting these DEGs into Ingenuity Pathway Analysis (IPA) and filtering pseudogenes, we identified 1165 analysis-ready molecules, with 579 downregulated and 586 upregulated molecules. Most DEGs were located in the cytoplasm (n = 348), followed by the nucleus (n = 210), plasma membrane (n = 202), and the extracellular space (n = 98). The location of the remainder of the DEGs was categorized as “other” (n = 307) (Figure 1B).

The location of most of the top 10 upregulated molecules was extracellular (molecule, fold change: CXCL-8, 53.229; CXCL-5, 12.520; CCL5, 5.857; CXCL-6, 5.433; CXCL-3, 4.306; and CST2, 4.225). The remainder of top upregulated molecules were located in the plasma membrane (major histocompatibility complex, class II, DR alpha (HLA-DRA, 150.402); CD74 molecule (CD74, 10.971); and solute carrier organic anion transporter family member 5A1 (SLCO5A1, 4.647).

Similarly, the location of the top ten downregulated molecules was most frequently either in the plasma membrane or was extracellular. The top downregulated molecule was potassium voltage-gated channel subfamily E regulator subunit 1 (KCNE1, −6.390), a plasma membrane ion channel, followed by glutamate ionotropic receptor kainate type subunit 4 (GRIK4, −4.471) also a plasma membrane ion channel. Top downregulated extracellular molecules included the enzyme ADP-ribosyltransferase 5 (ART5, −3.811) and interleukin 34 (IL-34, −3.407), a cytokine characterized as “other” that promotes monocyte and macrophage differentiation and viability via colony-stimulating factor-1 [25].

The remainder of the top 10 downregulated molecules included ankyrin repeat domain 45 (ANKRD45, −4.213), a nuclear protein involved in protein binding; IL6R antisense RNA 1 (IL6R-AS1, −4.159), an anti-sense RNA; PDZ domain containing 4 (PDZD4, −3.807), which has a cytoplasmic location and which is upregulated in synovial sarcomas [26]; senescence associated long noncoding RNA 2 (SALRNA2, −3.573), a noncoding RNA; and finally LIM domain only 3 (LM03, −3.353), a nuclear transcription coregulator.

#### 2.1.1. Extracellular Molecules

Given our focus on paracrine-mediated mechanisms by which HEMFs regulate epithelial and surrounding cell behavior, and that the top upregulated and downregulated molecules frequently had an extracellular location, we were particularly interested in the differential expression of these genes. Of the 98 molecules with an extracellular location; 54 were upregulated and 44 were downregulated. The general functional family of these molecules were Cytokines (n = 13), Enzymes (n = 8), Growth factor (n = 13), Peptidase (n = 10), Kinase (n = 2), Transporter (n = 3), and Other (n = 49) (Table S1).

The top upregulated cytokines were CXCL-8 (53.229), CXCL-5 (12.520), CCL-5 (5.857), CXCL-6 (5.433), and CXCL-3 (4.306). Downregulated cytokines were thyroid hormone receptor interactor 6, TRIP6 (−1.274) and CKLF-like MARVEL transmembrane domain containing 3 (CMTM3, −1.268).

The top upregulated growth factors were amphiregulin (AREG, 3.756), fibroblast growth factor 16 (FGF16, 2.609), endothelial cell specific molecule 1 (ESM1, 2.579), epiregulin (EREG, 2.465), and Dickkopf Wnt signaling pathway inhibitor 1 (DKK1, 1.758). Downregulated growth factors were jagged canonical Notch ligand 2 (JAG2, −1.697), bone morphogenetic protein 4 (BMP4, −1.618), latent transforming growth factor β binding protein 4 (LTBP4, −1.508), and vascular endothelial growth factor B (VEGFB, −1.235). We and others have previously shown that epithelial signals (e.g., Hedgehog ligands) modulate stromal [27], and more specifically, HEMF [23] BMP4 expression. LTBP4, like other LTBP isoforms, contributes to TGBβ activity and associates with fibrillin microfibrils [28].

Upregulated extracellular enzymes were cellular repressor of E1A-stimulated genes 2 (CREG2, 1.854), lipase G, endothelial type (LIPG, 1.648), and nudix hydrolase 6 (NUDT6, 1.576). The most downregulated extracellular enzyme was ADP-ribosyltransferase 5 (ART5, −3.811), which encodes a secretory protein that regulates target protein function via transfer of ADP-ribose [29].

The top upregulated proteases were matrix metalloprotease 1 (MMP1, 3.698), plasminogen like B2 (PLGLB1/PLGLB2, 2.602), chymotrypsinogen B1 (CTRB1, 2.170), serine protease 3 (PRSS3, 2.059), and pappalysin 1 (PAPPA, 1.710). Downregulated proteases included protein C, inactivator of coagulation factors Va and VIIIa (PROC, −2.265), matrix metallopeptidase 11 (MMP11, −1.595), serine protease 12 (PRSS12, −1.401), and MBL associated serine protease 1 (MASP1, −1.232). Stanniocalcin 1 (STC1, 3.422) and cysteine rich transmembrane BMP regulator 1 (CRIM1, −1.212) were up- and downregulated extracellular kinases, respectively. The most upregulated extracellular transporter was SEC23 homolog B, and COPII coat complex component (SEC23B, 1.431) and apolipoprotein D (APOD, −1.484) were downregulated.

Additional extracellular molecules described under the “other category” are listed in Table S1. Upregulated molecules in this category include extracellular members of the cystatin superfamily, the type 2 cystatins, cystatin SA (CST2, 4.225), and cystatin SN (CST1, 2.956). These are extracellular, secreted proteins that inhibit cysteine proteases [30]. Another upregulated molecule in this category was latent transforming growth factor beta binding protein 1 (LTBP1, 1.228), an extracellular protein that plays a role in TGF-beta regulation and also interacts with fibrillin-1 and fibronectin [28].

Several upregulated extracellular molecules in the “other” category were also from the serine protease inhibitor (SERPIN) gene family: serpin family D member 1 (SERPIND1, 2.433) and serpin family E members 1 and 2 (SERPINE1, 1.501 and SERPINE2, 1.304). SERPIND1 or heparin cofactor II (HCII) inhibits thrombin activity through interaction with heparin and promotes the release of leukocyte chemotactic factors and induces angiogenesis [31]. SERPINE1 or plasminogen activator inhibitor type-1 (PAI-1) is a major regulator of the urokinase plasminogen activator (uPA)-dependent pericellular plasmin-generating cascade with roles in stromal remodeling, cell growth, and migration [32]. It is induced by TGF-β1 and has increased expression in the basal epithelium of pediatric patients with active eosinophilic esophagitis (an allergen-mediated disease characterized by eosinophil recruitment to the esophagus [33]) and expression levels that correlate positively with subepithelial fibrosis [34]. More recently, PAI-1 has been shown to correlate with reduced esophageal compliance, while the inhibition of PAI-1 reduces primary human esophageal epithelial cell proliferation [35]. SERPINE2, unlike the other differentially expressed SERPINs is not circulating, but is expressed in a number of organs and/or cell types and impacts thrombus formation as well as cell proliferation via varied signaling pathways [36]. The most downregulated molecule in the “other” category was interleukin 34 (IL34, −3.407), which is a ligand for colony stimulating factor-1 receptor, the activation of which is critical for macrophage development [37]. R-spondin 2, a secreted glycoprotein and regulator of Wnt signaling [38], was also downregulated (RSPO2, −1.673).

#### 2.1.2. Plasma Membrane Molecules

Given that the ability to sense the environment could be critical to the function of this cell population, we also looked at the list of DEGs with location in the plasma membrane to identify potential mechanisms by which HEMFs detect noxious luminal stimuli encountered in GERD, transmit luminal signals, and interact with surrounding environment. The general functional family of these plasma membrane molecules were transporters (n = 34), transmembrane receptor (n = 34), G-protein-coupled receptor (n = 26), enzymes (n = 17), ion channel (n = 18), kinase (n = 9), phosphatase (n = 3), peptidase (n = 3), growth factor (n = 1), and other (n = 57) (Table S2).

Several genes encoding members of the solute carrier proteins (SLC) superfamily of plasma membrane transporter proteins were differentially expressed. These are a relatively understudied gene family that have been garnering increasing attention [39] especially in the GI tract [40]. Among the SLC superfamily members, the top upregulated transporter was a member of the solute carrier organic anion transporter (SLCO) family, solute carrier organic anion transporter family member 5A1 (SLCO5A1, 4.647). The SLCO proteins encoded by these genes are also known as organic anion transporting polypeptides (OATPs) that mediate the cellular uptake of organic ions including drugs [41,42]. The next most upregulated solute carrier gene in our dataset was solute carrier family 19 member 3 (SLC19A3, 2.546), responsible for the transport of thiamine [42]; and the most downregulated was solute carrier family 27 member 6 (SLC27A6, −2.756), a fatty acid transporter [43].

SLC expression in esophageal epithelial cells has recently been reported. Transporter genes with preferential expression in the esophagus (SLC2A1, SLC6A1, SLC6A9, SLC9A9, SLC15A2, SLC16A2, SLC16A6, SLC16A9, SLC16A14, SLC22A15, SLC28A3, SLC39A2, SLC42A3, SLC43A3, SLC44A5, and SLC03A1) as well as genes enriched in squamous epithelial suprabasal (SLC16A6, SLC16A9, SLC24A3, SLC42A3, SLC39A2, and SLCO2B1) and basal cell (SLC1A3, SLC1A4, and SLC7A2) populations have been identified [40].

Several plasma membrane ion channel molecules were also differentially expressed. Ion channels, also members of the family of transport proteins, maintain membrane potential via ion transport across cell membranes and participate in cellular excitation and signaling [42]. The top upregulated ion channel was potassium inwardly rectifying channel subfamily J member 15 (KCNJ15, 2.415), which is important in galvanotaxis or directional cell migration in response to weak electrical fields [44]; the most downregulated ion channel was potassium voltage-gated channel subfamily E regulatory subunit 1 (KCNE1, −6.390). TRPV1, a nonselective ion channel expressed by HEMFs and which partially regulates the HEMF response to acid [19] was not differentially expressed in acidic-bile-salt-treated HEMFs.

The top upregulated G-protein-coupled receptor was adhesion G-protein-coupled receptor F4 (ADGRF4, 2.274), followed by 5-hydroxytryptamine receptor 7 (HTR7, 1.853); adhesion G-protein-coupled receptor G1 (ADGRG1, 1.654); adhesion G-protein-coupled receptor L4 (ADGRL4, 1.612), and F2R like trypsin receptor 1 (F2RL1, 1.588). There was no differential expression of TGR5, a transmembrane G-protein-coupled bile acid receptor [45].

The top relevant upregulated transmembrane receptor was CD74 (CD74, 10.971), which is a cell-surface receptor for the cytokine macrophage migration inhibitory factor (MIF), whereby MIF-CD74 signaling activates prosurvival and proliferative pathways in wound healing; e.g., CD74 is upregulated during cutaneous wound healing [46]. This was followed by interleukin-7 receptor (IL-7R, 1.941) and triggering receptor expressed on myeloid cells 1 (TREM1, 1.938). The most downregulated transmembrane receptors included semaphorin 6A (SEMA6A, −2.098) and plexin C1 (PLXNC1, −2.015).

Ecto-5′nucleotidase (NT5E, 1.524), also known as CD73, was the top upregulated phosphatase located at the plasma membrane. NT5E has been reported on the surface of various cell types where it hydrolyzes extracellular adenosine monophosphate into inorganic phosphate and adenosine which can inhibit cellular immune responses [47]. Protein tyrosine phosphatase receptor type S (PTPRS, −1.299) was the most downregulated phosphatase. Of the plasma membrane peptidases, alanyl aminopeptidase membrane (ANPEP, 1.282) was upregulated and angiotensin 1 converting enzyme (ACE, −1.377) was downregulated. The most upregulated plasma membrane kinase was the EPH receptor B1 (EPHB1, 1.925) and the most downregulated was ephrin B3 (EFNB3, −2.129). Ephrin-EPH signaling regulates the developmental process involving morphogenesis, as well as cell differentiation, proliferation, and apoptosis. The signaling between EPH receptor tyrosine kinases and their membrane-tethered ligands, the ephrins, is also implicated in cancer, synaptic plasticity, and vasculogenesis [48].

#### 2.1.3. Validation of RNA Sequencing Genes with RT-PCR

**Figure 2:**
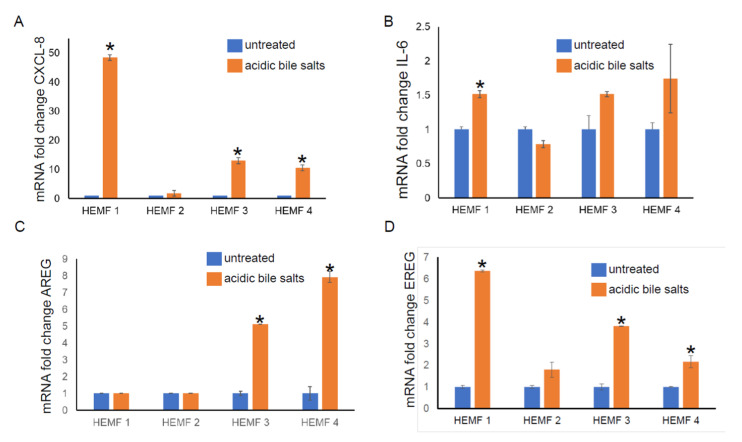
HEMF primary culture (HEMFs 1–4) mRNA fold change of (**A**) CXCL-8, (**B**) IL-6, (**C**) AREG, and (**D**) EREG. * = *p* ≤ 0.05. Results shown represent the means ± SEM of experiment conducted with primary cell cultures derived from 4 normal human esophagi.

To validate the differential expression of genes identified by RNA sequencing, we focused on the evaluation of the mRNA expression of differentially expressed, extracellular located cytokines, chemokines, and growth factors (CXCL-8, IL-6, AREG, and EREG) in our previously established primary HEMF cultures from normal human esophagus [19,22] treated with acidic bile salts. We then followed with an evaluation of the expression of these and additional genes in a previously established HEMF cell line [24]. The mRNA expression of CXCL-8 was elevated in three out of four primary cultures (Figure 2A). There was an increasing trend in the mRNA expression of IL-6 in three out of four primary HEMF cultures, with a statistically significant difference in primary culture established from HEMF 1 (Figure 2B). AREG mRNA was increased in two out of the four HEMF primary cultures (Figure 2C) and EREG was increased in three out of four primary HEMF cultures (Figure 2D).

**Figure 3:**
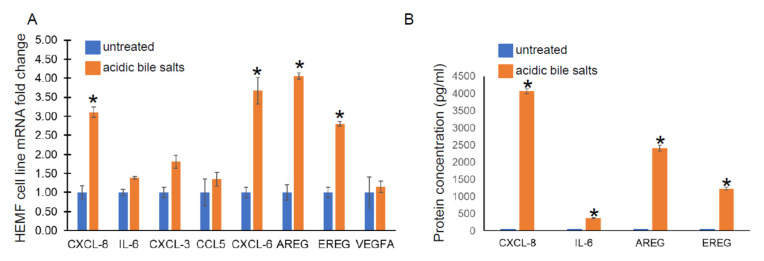
HEMF cell line mRNA expression and protein secretion. (**A**) mRNA expression of upregulated genes identified by RNA sequencing was evaluated in a HEMF cell line similarly treated with acidic bile salts and compared to untreated cells (grown in neutral serum-free myofibroblast media (SFMM; pH 7.2). mRNA expression of the following differentially expressed genes were evaluated: CXCL8, IL-6, CXCL3, CCL5, CXCL6, AREG, EREG, and VEGFA. Statistically significant differences are shown by * symbol with *p* value ≤ 0.05. Results shown represent the means ± SEM of three independent experiments conducted with HEMF cell line. (**B**) Proteins of genes identified by RNA sequencing were evaluated in supernatant of untreated and acidic-bile-salt-treated HEMFs. Statistically significant differences are shown by * symbol with *p* value ≤ 0.05. Results shown represent the means ± SEM of three independent experiments conducted with HEMF cell line.

Given its similar functional capacity and morphological properties to primary cultures, we utilized a previously established HEMF cell line treated with acidic bile salts, in a similar fashion to primary HEMFs, and assessed for the following genes from cytokine and growth factor families identified in our RNA-Seq data: CXCL-8, IL-6, CXCL-3, CCL5, CXCL-6, AREG, EREG, and VEGFA by RT-PCR. There was a significant increase in CXCL-8, CXCL-6, AREG, and EREG mRNA in the HEMF cell line treated with acidic bile salts, consistent with the DEGs observed in our RNA sequencing and consistent with HEMF primary cultures. CXCL-3, CCL5, and IL-6 showed nonsignificant trends toward an increase, (Figure 3A). We further validated the RNA sequencing data at the protein level by evaluating the supernatant for the secretion of the most differentially expressed chemokine and growth factor, CXCL-8 and AREG, respectively, as well as IL-6 and EREG (Figure 3B). We were able to detect significant quantities of CXCL-8, IL-6, as well as AREG and EREG in the supernatant of HEMFs treated with acidic bile salts compared to untreated HEMF supernatant.

**Figure 4:**
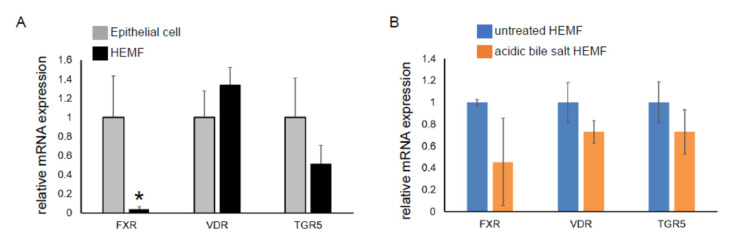
mRNA expression of bile acid receptors in a squamous epithelial cell line and in a HEMF cell line. mRNA expression of bile acid receptors TGR5, VDR, and FXR were evaluated in (**A**) untreated epithelial cells and HEMFs and in (**B**) untreated vs. acidic-bile-salt-treated HEMFs. Statistically significant differences are shown by * symbol with *p* value ≤ 0.05. Results shown represent the means ± SEM of three independent experiments conducted with a HEMF cell line and with an esophageal epithelial cell line.

We had previously shown that the HEMF response to acidified media was partially mediated via TRPV1, a nonselective ion channel [19]. Mechanisms by which HEMFs respond to a mixture of acidic bile salts remained unexplored. We therefore evaluated the expression of the nuclear bile acid receptors FXR [49,50] and VDR [51] and the transmembrane bile acid receptor TGR5 [52] in a human esophageal epithelial cell line with basal cell phenotype. The mRNA expression of VDR and TGR5 in HEMFs was similar to that in squamous epithelial cells, while the mRNA expression of FXR was markedly less in HEMFs (Figure 4A). Consistent with RNA-Seq, there was no change in HEMF expression of these bile acid receptors in response to treatment with acidic bile salt (Figure 4B).

### 2.2. Functional Analysis of DEGs

#### 2.2.1. Identification of Canonical Pathways Activated in Acidic-Bile-Salt-Treated HEMFs

**Figure 5:**
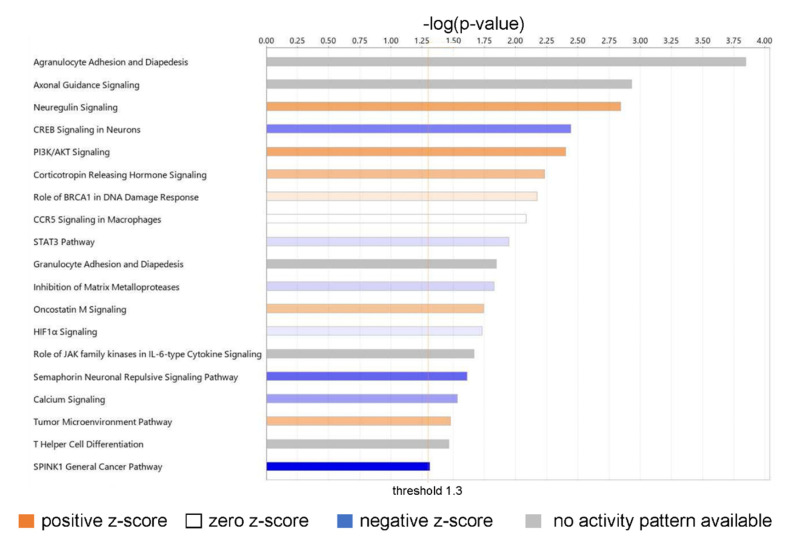
Canonical pathways of biological significance activated in our dataset. Significance values are displayed as −log of the *p*-value, calculated by the right-tailed Fisher’s exact test and which indicates the probability of association of molecules from our dataset with the canonical pathway by random chance alone. All pathways shown have −log (*p*-value) > 1.3 or *p* < 0.05.

An Ingenuity Pathway Analysis of the DEGs from RNA-Seq identified the top canonical pathways of biological significance activated in our dataset (Figure 5).

The top biologically relevant significant canonical pathway enriched by our molecules was Agranulocyte Adhesion and Diapedesis (18 molecules, z-score NaN, −log (*p*-value) 3.845, ratio 18/100 = 0.18). Nine of the eighteen molecules included in this pathway had an extracellular space location and were either cytokines (CXCL-8, CXCL-5, CCL5, CXCL-6, CXCL-3, IL-33) or proteases (MMP1, MMP3, MMP11). Six molecules in this pathway were located in the plasma membrane and were transmembrane receptors (ITGA1, CLDN4, ITGA5, ITGA6), a kinase (PODXL), or “other” (CLDN11) (Table S3).

Neuregulin Signaling was also identified as a top canonical pathway (15 molecules, z-score 1.667, −log (*p*-value) 2.842, ratio 15/92 = 0.163) (Table S3). Two molecules in this pathway were located in the extracellular space and were growth factors (AREG, EREG); six molecules were located in the cytoplasm and were enzymes (HSP90AA1, HSP90AB1, PLCG2) or “other” (ERRFi1, HSP90B1, TMEFF2); two molecules were located in the nucleus and were kinases (CDK5, CDKN1B); and five were located in the plasma membrane and were an enzyme (RAP2B), a growth factor (NRG1), or transmembrane receptors (ITGA2, ITGA5, ITGA6) (Table S2). Neuregulins (NRGs) are part of a large family of EGF-like signaling molecules involved in cell–cell communication [53]. They are ligands for receptor tyrosine kinases of the ErbB (epidermal growth factor) family (ErbB2, ErbB3, and ErbB4).

Several additional significant pathways with roles in inflammation and immune cell signaling were also identified, including PI3K/AKT signaling (21 molecules, z-score 1.667, −log (*p*-value) 2.403, ratio 21/163 = 0.129); CCR5 Signaling in Macrophages (10 molecules, z-score 0, −log (*p*-value) 2.085, ratio 10/61 = 0.0164); STAT3 pathway (15 molecules, z-score 0.378, −log (*p*-value) 1.947, ratio 15/114 = 0.132); Granulocyte Adhesion and Diapedesis (12 molecules, z-score NaN, −log (*p*-value) 1.845, ratio 12/86 = 0.14); Role of JAK family kinases in IL-6-type Cytokine Signaling (five molecules, z-score NaN, −log (*p*-value) 1.666, ratio 5/24 = 0.208); and HIF1α Signaling (19 molecules, z-score 0.229, −log (*p*-value) 1.73, ratio 19/165 = 0.115); and T Helper Cell Differentiation (six molecules, z-score NaN, −log (*p*-value) 1.463, ratio 6/36 = 0.167).

#### 2.2.2. Diseases and Biological Functions

**Figure 6:**
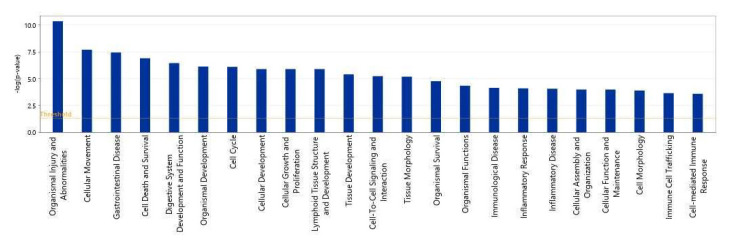
Top biological processes impacted by acidic bile salt treatment of HEMFS. Significance values are displayed as −log of the *p*-value, calculated by the right-tailed Fisher’s exact test and which measures the likelihood of association between molecules in our dataset and a pathway by random chance alone. Threshold denotes −log (0.05) = 1.3; taller bars equate to increased significance.

We then used IPA to show which biological processes or diseases were impacted by acidic-bile-salt-treated HEMFs. The topmost relevant processes included organismal injury and abnormalities, a number of cellular processes including cellular movement, cell death and survival, cellular growth and development, and immune cell trafficking and cell-mediated response were also involved (Figure 6).

#### 2.2.3. Upstream Analysis: Identification of Upstream Regulators and Causal Networks

The upstream regulator analysis (URA) tool in IPA identified 1000 upstream regulators with *p* ≤ 0.05 that based on available literature could be responsible for driving gene expression changes in our dataset as well as the associated affected diseases and biological functions.

**Figure 7:**
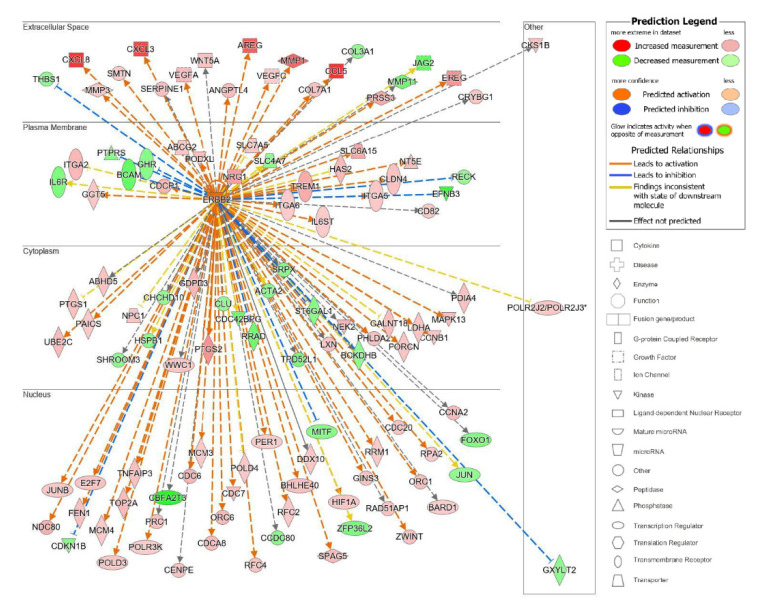
Top upstream regulator ErbB2. ErbB2 and the downstream targets in our dataset are displayed with their subcellular location. The upstream regulator analysis (URA) tool was used in IPA to identify the top upstream regulators that based on available literature could be responsible for driving expression changes seen in our dataset and that met criteria for *p* < 0.05. The top upstream regulator based on *p*-value of overlap was epidermal growth factor receptor 2 (ErbB2, *p* = 3.28 × 10^−16^). * indicates multiple identifiers in the dataset file map to a single gene in the Global Molecular Network.

The top upstream regulator overall based on the *p*-value of the overlap was a kinase located in the plasma membrane, epidermal growth factor receptor 2 (ErbB2), also known as HER2 and Neu (Table S4). ErbB2 is a member of the epidermal growth factor family of receptor tyrosine kinases, which also includes ErbB1, also known as EGFR, and ErbB3. Unlike the other family members, ErbB2 does not have known ligands and can respond to extracellular stimuli once it has formed a heterodimer with either ErbB1 or ErbB3 [54]. Although ErbB2 itself was not differentially expressed, the information from the IPA knowledge bases indicated that ErbB2 as a regulator targeted a significant set of analyzed molecules in our dataset and that its influence could be predicted by the changes seen in our dataset. The predicted state of ErbB2 was that of activation, with a significant z-score of 5.716. There were 114 target molecules in our dataset molecules regulated by ErbB2, with 69 of the 114 genes with a measurement direction consistent with the activation of ErbB2 (Table S5). The activation of ErbB2 was postulated to causally lead to the up- and downregulation of genes in our dataset, including the differential expression of molecules located in the extracellular space, plasma membrane, cytoplasm, and nucleus (Figure 7). Extracellular targets upregulated in response to being treated with acidic bile salts included CXCL-8, AREG, and EREG. Downregulated extracellular targets included COL3A1, JAG2, THB1, and MMP11.

The remaining nine top upstream regulators were CG (a complex), followed by TNF (a cytokine), TGFB1 (a growth factor), IGF1 (a growth factor), HGF (a growth factor), VEGF (a group), EGFR or ErbB1 (a kinase), EGF (a growth factor), and Pkc(s) (group) (Table S4).

**Figure 8:**
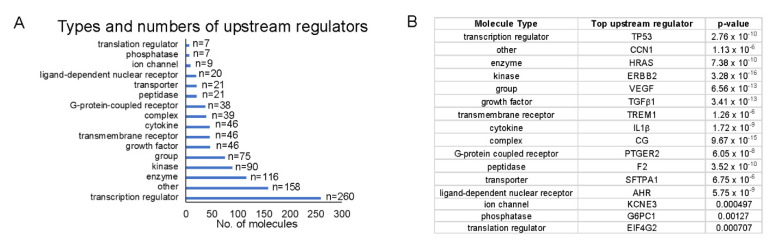
Top upstream regulators. (**A**) The number of each upstream regulator type is shown and (**B**) within each type of upstream regulator, we identified the most significant upstream regulator (*p*-values shown). Upstream regulators were most frequently transcription regulators. The most significant transcription regulator and transmembrane receptor were TP53 and TREM1, respectively.

Because we were interested in identifying upstream regulators that could provide a pathway by which HEMFs responded to stimulation, we quantified upstream regulators by type (Figure 8A) and identified the top upstream regulators of each type, focusing on those that were located in the plasma membrane (e.g., as was ErbB2) or classified as transmembrane receptors or G-protein-coupled receptors (Figure 8B).

**Figure 9:**
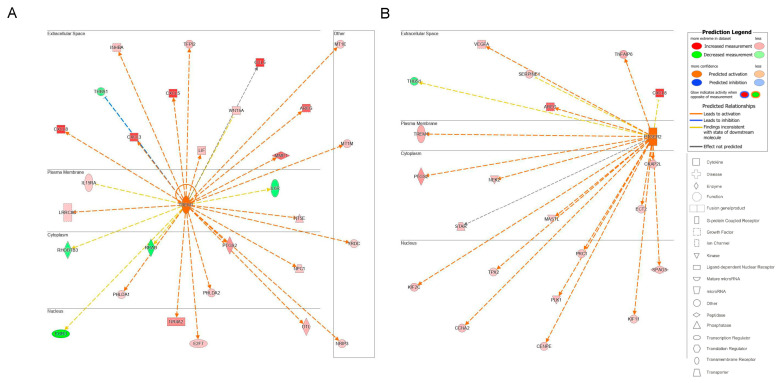
Upstream regulators identified in IPA. The subcellular location of associated target molecules of the upstream regulators (**A**) TREM1, a transmembrane receptor (*p* = 1.26 × 10^−6^) and for (**B**) PTGER2, a G-protein-coupled receptor (*p* = 6.05 × 10^−8^) are shown. The differential expression of associated downstream target molecules identified in our dataset is shown (red is increased, green is decreased) as is their subcellular location.

The top transmembrane receptor that was an upstream regulator, triggering receptor expressed on myeloid cells 1 (TREM1) had 29 target molecules (*p* = 1.26 × 10^−6^) (Figure 9A). The activation of TREM1, is postulated to causally lead to the up- and downregulation of the genes in our dataset, located in the extracellular space, plasma membrane, cytoplasm, and nucleus. Similar to the upstream regulator ErbB2, extracellular targets of the upstream regulator TREM1 with increased differential expression in response to treatment with acidic bile salts included a number of chemokines and growth factor, including CXCL-8 and AREG.

Several costimulatory molecules involved in immune regulation were also identified as transmembrane upstream regulators including CD40 (activation z-score: 2.127; *p*-value of overlap: 0.000379), CD36 (activation z-score: 2.889; *p*-value of overlap: 0.000744), CD74 (activation z-score: N/A; *p*-value of overlap: 0.00301), and CD28 (activation z-score 3.882; *p*-value of overlap: 0.00892).

Toll-like receptor (TLR) TLR-4, which we have previously shown are expressed by HEMFs [19] was also identified as a transmembrane upstream regulator (activation z-score: 2.293; *p*-value of overlap: 0.0267). Other TLRs identified as upstream regulators included TLR1 (activation z-score: N/A; *p*-value of overlap: 0.0205), TLR2 (activation z-score: 3.487; *p*-value of overlap: 0.000155), TLR3 (activation z-score: 3.157; *p*-value of overlap: 0.0265), TLR5 (activation z-score: 2.183; *p*-value of overlap: 0.00492), TLR7 (activation z-score: 2.788; *p*-value of overlap: 0.00834), and TLR9 (activation z-score: 3.014; *p*-value of overlap: 0.0222).

The top G-protein-coupled receptor that was an upstream regulator was prostaglandin E receptor 2, (PTGER2), which had 21 target molecules (*p* = 6.05 × 10^−8^). The activation of PTGER2 was postulated to causally lead to the up- and downregulation of genes in our dataset, located in the extracellular space, plasma membrane, cytoplasm, and nucleus. Extracellular targets with increased differential expression included AREG and CXCL-8 (Figure 9B).

**Figure 10:**
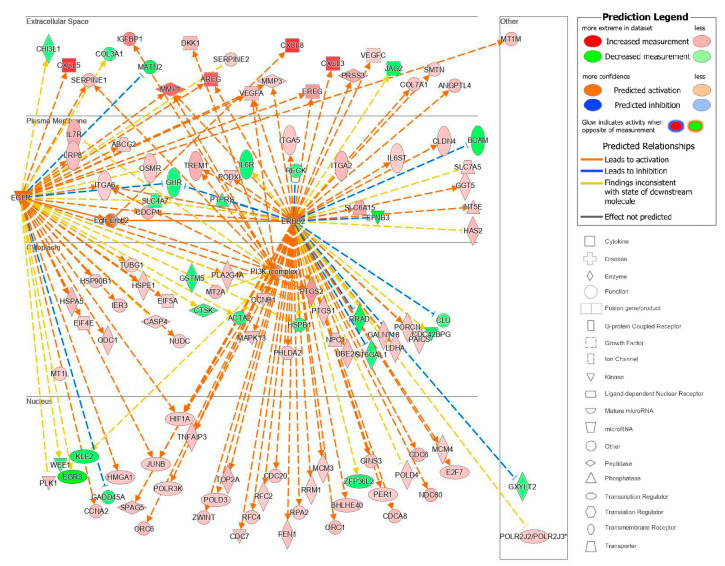
The Egfr–ErbB2 causal network. EGFR–ErbB2, a complex, was a master regulator with intermediate regulators EGFR, ErbB2, and PI3K (network-bias-corrected *p*-value 0.0001). The subcellular location and expression (red is increased, green is decreased expression) of these master and intermediate regulators and the most differentially expressed downstream target molecules in our dataset are shown. * indicates multiple identifiers in the dataset file map to a single gene in the Global Molecular Network.

The upstream analysis also identified causal networks to identify master upstream regulators that could act directly on the data set molecules or through an intermediate regulator. We focused on those master upstream regulators with a plasma membrane location and with the most significant *p*-values of overlap after correcting for the network bias that can be introduced by molecules connected to many upstream regulators The top master regulator with a plasma membrane location was a kinase complex, Egfr–ErbB2 (Figure 10). From our analyzed data set, 115 target molecules were in this causal network and 4 upstream regulators participated. Moreover, 87 of the 115 genes had a measurement direction consistent with the activation of Egfr–Erbb2. This causal network of master regulators that are cell surface receptors was interesting because it also identified a potential pathway of communication between HEMFs and the extracellular space. Overall, the identification of these upstream and causal regulators highlight potential mechanisms by which HEMFs respond to noxious luminal- and epithelial-derived stimuli.

#### 2.2.4. Inhibition of HEMF-Derived CXCL-8, but Not HEMF-Derived AREG Inhibits Squamous Epithelial Cell Proliferation

**Figure 11:**
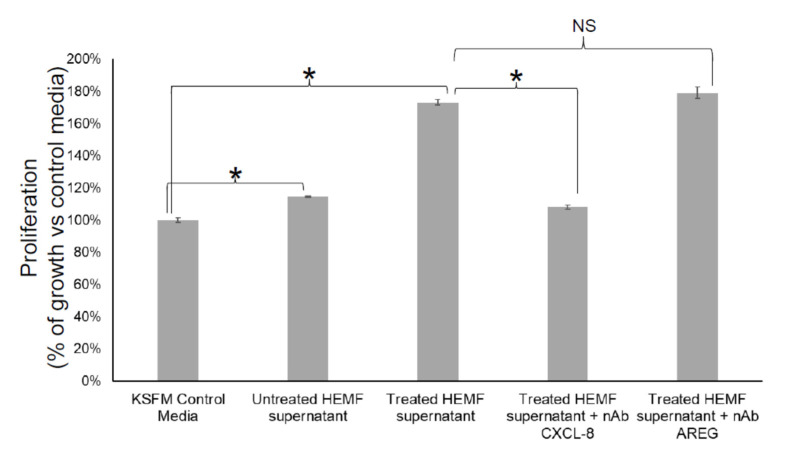
Epithelial cell proliferation in response to inhibition of HEMF-derived factors. Epithelial cells (2000 cells/well of 96-well plate) were treated with KSFM (epithelial cell media) or supernatant of untreated and acidic-bile-salt-treated HEMFs (in background KSFM media overnight) in the presence and absence of neutralizing antibodies for CXCL-8 and AREG. Results shown represent the means ± SEM of three independent experiments conducted with a HEMF cell line * *p* < 0.05; NS, nonsignificant with ANOVA with multiple comparisons.

We next considered whether HEMF-secreted factors identified by differential expression in RNA-Seq and as targets of the most significant upstream regulators were responsible for biological processes such as cellular growth and proliferation, including basal cell hyperplasia observed in GERD [23]. We evaluated the proliferation of an immortalized squamous epithelial cell line with a predominantly basal cell phenotype [55,56] in response to the HEMF supernatant in the presence of neutralizing antibodies for CXCL-8 and AREG, the top differentially expressed gene and growth factor, respectively (Figure 11). Consistent with our previous work [22,23], epithelial cell proliferation increased in the presence of the supernatant from untreated HEMFs (14%, *p* < 0.05). Moreover, we also showed that epithelial proliferation was even more profoundly increased in the presence of the supernatant from acidic-bile-salt-treated HEMFs (73%, *p* < 0.05). This increase in proliferation was inhibited in the presence of neutralizing antibodies for CXCL-8. The inhibition of the HEMF-secreted growth factor AREG in the HEMF supernatant did not have an effect on epithelial proliferation.

## 3. Discussion

In this study, for the first time, we utilized RNA sequencing to identify activated pathways in primary cultures of HEMFs treated with components of noxious luminal insults encountered in GERD [9,57]. HEMFs encounter these luminal factors (e.g., acidic bile salts) in the setting of an impaired epithelial barrier present in GERD. We showed that most of the top differentially expressed molecules in response to treatment with acidic bile salts were either extracellular or located in the plasma membrane. This observation highlights the paracrine contribution of HEMFs [23] and also, perhaps, their capacity to sense and respond to their environment. We had previously shown that HEMFs expressed the acid receptor TRPVI and now we also showed the mRNA expression of bile acid receptors. A further analysis showed that HEMF treatment with acidic bile salts activated a number of inflammatory, proliferative, and immune-cell-mediated pathways and identified CXCL-8 as a HEMF-derived chemokine that directly regulates basal cell proliferation observed in GERD. Consistent with prior work [22,23] which showed that the effect of HEMFs on overlying squamous epithelium was mediated in part by paracrine mechanisms, the top differentially expressed molecules in treated HEMFs in our study were extracellular. As such, we proceeded to validate the most differentially expressed cytokines, chemokines, and growth factors in previously established HEMF primary cultures [19,22] used for RNA sequencing in this study and also in a previously validated HEMF cell line [24] treated with acidic bile salts.

The analysis of our RNA-Seq data suggested that HEMF treatment with acidic bile salts had a significant impact on a number of cellular processes including growth and proliferation. Our observation that treatment of a squamous epithelial cells with a supernatant from acidic-bile-salt-treated HEMFs led to an increase in proliferation confirmed prior work that HEMF-secreted factors could drive epithelial changes in the GERD environment [23]. We previously showed that HEMF-secreted factors in response to acid and acidic bile salts increased epithelial thickness [23]. Histological changes in GERD include basal cell hyperplasia and an increase in epithelial thickness [58]. The RNA-Seq data identified CXCL8 and AREG as possible candidate mediators and the increase in gene and protein expression was validated. To determine whether these HEMF-derived factors contributed to the basal cell hyperplasia observed in GERD, we evaluated the proliferation of an epithelial cell line with basal phenotype [55] in the presence of neutralizing antibodies for CXCL-8 and AREG. As discussed below, CXCL-8 inhibition from HEMFs, but not from AREG, inhibited epithelial cell proliferation in this model.

Despite the expected variability between primary cultures in degree of mRNA fold change, the pattern of response was similar across cultures with the most differentially expressed gene being CXCL-8. Consistent with RNA sequencing results and the primary cultures, we also confirmed a several fold increase in mRNA expression and protein secretion of CXCL-8 in our previously described immortalized HEMF cell line treated with acidic bile salts [24]. CXCL-8 or IL-8 is a proinflammatory chemokine implicated in GERD-associated mucosal inflammation [59]. It is also a chemokine that plays an essential role in the activation and trafficking of immune cells, particularly neutrophils [60]. CXCL-8 mediates its effects via extracellular binding to C-X-C chemokine receptor type 1 (CXCR1) and C-X-C chemokine receptor type 2 (CXCR2), two G-protein-coupled receptors [60] expressed by squamous epithelial cells. CXCL-8 also plays a role in promoting proliferation and inhibiting apoptosis [61] and promoting epithelial–mesenchymal transition [62]. The inhibition of CXCL-8 in the HEMF supernatant profoundly decreased squamous epithelial proliferation, suggesting an important role for HEMF-derived chemokines. It remains to be seen whether these findings can be reproduced in 3D organotypic and organoid models, which better reflect the complexity of human esophageal mucosa.

RNA sequencing and qRT-PCR with the HEMF cell line also showed an increase in another chemokine in the CXC class, CXCL6 or human granulocyte chemotactic protein 2 (GCP-2). CXCL-6/GCP-2 is expressed by a variety of epithelial cells, macrophages, and mesenchymal cells of different tissues during inflammation, where it serves as a chemoattractant for leukocytes and also plays a role in the activation of endothelial cells [63]. Histologic changes in GERD have been reported to be driven by a predominantly T-cell-mediated inflammation that begins in the submucosa [8,12]. We have previously shown an increase of α-SMA+CD31+ endothelial cells in GERD biopsy samples vs. normal esophagus [19]. CXCL-6 also has pro-proliferative effects [64] including promoting the proliferation, migration, and invasion of esophageal squamous cell carcinoma cells [65]. Like CXCL-8, its effects are mediated by interacting with CXCR1 and CXCR2 [60], which are expressed on a variety of immune cells as well as squamous epithelial cells [66]. Our present work shows that the expression of this chemokine is also upregulated in HEMFs in response to acidic bile salts.

We had previously shown an increase IL-6 mRNA expression in primary HEMFs treated with acidified media alone [19]. We were curious whether this increase would be reproduced with acidified bile salts, which better reflect refluxate encountered in GERD [9,57] patients. While RNA sequencing did not demonstrate a differential expression of IL-6 in HEMF primary cultures treated with acidified bile salts, there was certainly a trend (Table S1). qRT-PCR performed on HEMF primary cultures used for RNA-Seq also showed a trend in IL-6 mRNA in three out of four primary cultures, and a significant increase in one primary culture (HEMF 1). Similarly, there was a trend in increase in IL-6 mRNA expression in a HEMF cell line treated with acidic bile salts. The evaluation of the HEMF supernatant demonstrated a clear increase in IL-6 protein secretion from acidic-bile-salt-treated HEMFs. Temporal factors and the timing of mRNA versus protein evaluation may be a potential explanation for the apparent discrepancy between IL-6 mRNA expression and the observed increased in IL-6 protein secretion [67,68]. While RNA sequencing showed a differential expression of leukemia inhibitory factor (LIF, 1.475), there was no differential expression of other IL-6 family members (IL-11, IL-27, oncostatin M (OSM), ciliary neurotrophic factor (CNTF), cardiotrophin 1 (CT-1), and cardiotrophin-like cytokine factor 1 (CLCF1)). Overall, these findings suggest, as expected, that the gene expression profile of acid-treated HEMFs likely differs from acidified-bile-salt-treated HEMFs. Curiously, we previously showed that the phenotype of increased thickness of the squamous epithelium in the 3D-OTC model with supernatant established from acid- and acidified-bile-salt-treated HEMFs was similar [23], suggesting that the factors responsible for the increase in squamous epithelial thickness seen with these two conditions are due to a secreted factor common to both treatment conditions.

We also observed an increase in expression and secretion of two epithelial growth factor receptor (EGFR) ligands, AREG and EREG in HEMF primary cultures and HEMF cell line. There was no change in the other EGFR ligands, EGF, transforming growth factor-alpha (TGFA), heparin-binding EGF-like growth factor (HBEGF), betacellulin (BTC), and epigen (EPGN) [69]. AREG can stimulate the proliferation of most cells via its binding to EGFR, a widely expressed transmembrane tyrosine kinase, and the activation of intracellular signaling cascades regulating cell survival, proliferation, invasiveness, motility, and angiogenesis such as MAPK, PI3/AKT, STAT, and mTOR. AREG can also induce the activation of ErbB2, ErbB3, and ErbB4 after binding to EGFR [70]. AREG expression is induced in a number of cell types including cervical, airway, bronchial, gastric, and intestinal epithelial cells, fibroblasts, hepatocytes, T cells, macrophages, and salivary gland epithelial cells, by a variety of factors including IL-8 [70]. AREG has also been previously reported to be increased in a human colorectal cancer cell line treated with secondary bile acids or salts such as deoxycholic acid but not the primary bile acid cholic acid [70,71]. Our acidic bile salt mixture consisted of conjugated primary bile acids.

EREG is also expressed in a wide variety of tissues with increased expression in inflammatory states where it contributes to inflammation, wound healing, and tissue repair through a number of mechanisms including angiogenesis, vascular remodeling and cell proliferation. These processes are driven by numerous signaling cascades including the Raf/Ras/MAP kinase (Erk) signaling pathway, the phospholipase C gamma pathway, and the P13 kinase/Akt signaling pathway [72]. To our knowledge, neither AREG nor EREG expression and secretion has been examined in human esophageal myofibroblasts. The inhibition of AREG in our HEMF supernatant did not decrease epithelial proliferation, however, in the MTT assay.

Finally, due to the limited availability of primary culture samples, we looked at the expression of several other chemokines and growth factors highlighted by RNA sequencing (CXCL-3, CCL5, VEGFA, and CXCL-6) in our previously validated HEMF cell line alone. We observed a more than threefold increase in CXCL6 mRNA but were unable to confirm an increase in CCL5 or VEGFA mRNA in the HEMF cell line. There was a trend toward an increase in CXCL-3. Overall, these findings corroborate the overall consistency of results between RNA sequencing, HEMF primary cultures, and the HEMF cell line. As expected, given our use of primary cultures, the transcriptome difference was dominated by subject, although we still observed the effect of treatment with acidic bile salts. Primary cultures are expectedly subject-dominated, as seen in the heterogeneity of fold changes observed with qRT-PCR performed in our primary HEMFs. Consistent with the differential expression of chemokines identified in our dataset, a number of inflammatory and immune-cell-mediated canonical pathways were identified by evaluation of differentially expressed genes in our dataset. These included agranulocyte and granulocyte adhesion and diapedesis, CCR5 signaling in macrophages, and T helper cell differentiation.

The differential expression of a number of growth factors in the EGF family, is also consistent with the upstream and causal regulators identified by our analysis that suggest a prominent role in the epidermal growth factor receptor (EGFR)/ErbB pathway for HEMF activation in response to noxious stimuli encountered in GERD. Previous studies in different tissue types have suggested that acids and bile acids can activate EGFR in epithelial cells lines via a multistep process that involves the ligand-independent transactivation of cytoplasmic kinases followed by the phosphorylation of intracytoplasmic tyrosine residues of the EGFR molecule, and/or possibly via the activation of dormant EGFR ligands [73]. Although there was no differential expression change of EGFR or ErbB2 themselves, molecular changes observed in our dataset could be driven by modifications to these molecules beyond RNA expression (e.g., post-translational modification). The activation of this pathway may also be responsible for driving the observed differential expression of factors with chemotactic properties for immune cells. These findings suggest, that in addition to the previously described HEMF-epithelial interactions in the human esophagus in GERD, there may also be a role for HEMF contribution to the immune-mediated pathogenesis of GERD, including the observed increase in lymphocytes seen in esophagitis [12].

We have previously shown that the HEMF response to acidified media may be partially mediated via the TRPV1, a nonselective ion channel [19]. Mechanisms by which HEMFs respond to a mixture of acidic bile salts remain unexplored. Both cell surface (TGR5) [52] and nuclear bile acid receptors (VDR, FXR) [49,50,51] have been evaluated in esophageal mucosa, and their expression has been described in the basal layer of the esophageal squamous epithelium [51,74,75]. We showed that the mRNA expression of the nuclear receptor VDR and the transmembrane receptor were similar in an epithelial cell line and in HEMFs, while the expression of the nuclear receptor FXR was markedly less in HEMFs. Consistent with our RNA-Seq data set, we were unable to detect a change in expression of any of these receptors in response to treatment with acidic bile salts. Additional studies are needed to determine whether these bile acid receptors are critical to the observed responses in HEMFs, in particular the transmembrane G-protein-coupled receptor TGR5, which, unlike the intracellular nuclear receptors, does not depend on transport systems for cellular ligand uptake [45].

The limitations of the current study include a possible overinterpretation of RNA seq data, especially given the evidence of several regulatory mechanisms that are in play after the manufacture of mRNA [76]. We also did not address the role of transcript variants which can affect the function of gene products [77]. In addition, gene expression does not necessarily reflect protein abundance, especially in large-scale data sets, or as mentioned earlier, the role of post-translational modifications [76]. We attempted to validate the RNA-Seq data with qRT-PCR on samples of HEMF primary cultures and a cell line, following through with an evaluation of protein data, and attempted to show the functional relevance of the identified HEMF-derived mediators through inhibition studies. Thus far, we investigated the effect of identified HEMF-derived mediators only on epithelial proliferation. AREG did not have an effect on epithelial proliferation using the MTT assay; other EGFR ligands such as EREG were not evaluated. This work sets the stage for future studies to interrogate the role of HEMF-derived factors in complementary 3D-organotypic and organoid cultures that allow for the evaluation of epithelial proliferation/differentiation and barrier integrity.

In conclusion, we demonstrated that HEMFs treated with acidic bile salts, as a model of GERD, activated several inflammatory and/or proliferative pathways that involve paracrine mediators. These mediators could potentially have autocrine effects as well as paracrine effects. We demonstrated that HEMF-derived CXCL-8 had direct effects on squamous epithelial proliferation. Cell–cell signaling, including the effects of HEMFs on surrounding nonimmune stromal cells, immune cells, and adjacent epithelial cell differentiation and gene expression in the esophagus, warrants continued investigations.

## 4. Materials and Methods

### 4.1. Cell Culture

Previously characterized primary HEMF cultures, established from surgical resections of deidentified normal human esophagi [19] (HEMFs 1–4) and a previously established immortalized HEMF cell line with phenotypic, genotypic, and functional similarity to primary HEMFs [24] were cultured in HEMF growth media, comprised of Dulbecco’s minimum essential media (DMEM) (Life technologies, cat# 11965118, Carlsbad, CA, USA) with 10% fetal bovine serum (Sigma, cat# F0926-500ml, St. Louis, MO, USA) and supplemented with 10 mg/mL insulin (Sigma: cat#I9278, St. Louis, MO, USA), 2 ng/mL human EGF (Tonbo Biosciences, cat# 21-836-U100, San Diego, CA, USA), 10 µg/mL transferrin (Sigma, cat# T8158), and 10 µg/mL gentamycin (Sigma, cat# G1397). Cells were incubated at 37 °C with 5% CO_2_.

A human esophageal epithelial cell line (kind gift of the Rustgi lab) was cultured as previously described in KSFM [23].

### 4.2. HEMF Treatment with Acidic Bile Salt Mixture

HEMFs were grown in 6-well plates and treated for 8 min, every 2 h, a total of 4 times over the course of one day with the following conditions: serum-free HEMF growth media (pH 7.2) as the control or serum-free HEMF media supplemented with an acidic bile salt mixture of conjugated primary bile acids (glycocholic, taurocholic, glychchenodeoxycholic, and taurochenodeoxycholic-, glycodeoxycholic-, and taurodeoxycholic-acids (20:3:15:3:6:1 molar concentration, final concentration 400 µM)) adjusted to pH 4.5 with 1 N HCl as per [78]. Between treatments, cells were incubated in full HEMF media. After each treatment, cells were washed twice with 1X PBS and incubated in serum-free myofibroblast media (pH 7.2). Cells were harvested the next day for RNA and the supernatant collected for ELISA. In cases where the supernatant was to be used for the treatment of epithelial cells, HEMFs were grown overnight in keratinocyte serum-free media (KSFM). Pilot studies showed that HEMFs tolerated growth in KSFM for up to 48 h without significant effects on viability or morphology.

### 4.3. RNA Sequencing Expression Profiling and Pathway Analysis of Gene Networks

Total RNA was isolated from primary HEMF cultures (n = 4) using GeneElute Mammalian Total RNA miniprep Kit (Millipore Sigma, cat# RTN70-1KT, Carlsbad, CA, USA) per manufacturer’s recommendations and submitted for RNA sequencing (Next Seq500 High Output sequencing, UCLA Technology Center for Genomics & Bioinformatics (TCGB) Laboratory/UCLA sequencing core). KAPA Stranded RNA-Seq Kit (Roche) poly-A kit was used for the mRNA sequencing library preparation. All libraries were sequenced single-end 75 nt on Illumina NextSeq 500 with a target coverage of 30 million reads. RNA-Seq data was analyzed using the RNA-Seq workflow in Partek Flow software (v7. Partek Inc., St. Louis, MO, USA). Raw sequencing reads were trimmed based on their quality scores (minimum Phred score 20, minimum read length 25 nt from both end) before being mapped to the human genome hg38 using STAR version 2.7.3a with default parameter settings. Aligned reads were then quantified to Gencode annotation release 34 using Partek’s E/M method and genes with fewer than 10 reads in all samples were excluded from the subsequent analyses. Gene counts were normalized using the upper quartile normalization (Bullard et al., 2010) before being subjected to a differential expression analysis using a 2-way ANOVA method. Differentially expressed genes (DEGs) were selected using as criteria a *p*-value < 0.05 and absolute fold change >1.2.

We applied QIAGEN’s Ingenuity^®^ Pathway Analysis tool to our DEGs (IPA^®^, QIAGEN Redwood City, CA, USA, www.qiagen.com/ingenuity, accessed on 20 November 2020). Significance values for canonical pathways are displayed as −log of the *p*-value, calculated by the right-tailed Fisher’s exact test and which indicates the probability of association of molecules from our dataset with the canonical pathway by random chance alone. The ratio refers to the number of molecules in each pathway that meets our cutoff criteria (*p* < 0.05 and fold change ≥1.2 or ≤−1.2) divided by the total number of molecules that constitute the pathway and that are in the reference set. The z-score is a statistical tool used by IPA to assess the predictions of the activation or inhibition of the pathway based on available literature, where z-scores ≥ 2 are considered statistically significant. When available, the z-scores are noted or shown. Longer bars in figures equate to increased significance. The Diseases & Functions Analysis identified the biological functions and/or diseases that were most significant from the data set. Molecules from the dataset that met the *p*-value cutoff of <0.05 and absolute fold change cutoff of >1.2 and were associated with biological functions and/or diseases in the QIAGEN Knowledge Base were considered for the analysis. A right-tailed Fisher’s Exact Test was used to calculate a p-value determining the probability that each biological function and/or disease assigned to ourdata set is due to chance alone.

### 4.4. RNA Isolation and RT-PCR

Total RNA was isolated using the GeneElute Mammalian Total RNA miniprep Kit (Millipore Sigma. Cat# RTN70-1KT, Carlsbad, CA, USA) per the manufacturer’s recommendations. cDNA was synthesized using random primers (Life technologies, cat # 48190011, Carlsbad, CA, USA) and were amplified using SYBR green gene expression master mix (Life Technologies, cat #87893879, Carlsbad, CA, USA). To validate RNA sequencing gene expression data, we selected several upregulated and downregulated genes associated with inflammation and proliferation and validated them by qRT-PCR. Genes assessed for mRNA fold change were CXCL-8, IL-6, CXCL-3, CXCL-6, CCL5, VEGFA, BMP4, AREG, and EREG. Human 18s was used as the endogenous control to normalize the samples using the ΔΔCT method of relative quantification, where CT is the threshold cycle as previously described [22]. mRNA expression was also determined for bile acid receptors VDR, FXR, and TGR5. Briefly, total RNA was converted to complementary DNA and used for qRT-PCT as previously described [22].

### 4.5. ELISA

The HEMF supernatant was collected for evaluation of CXCL-8, IL-6, AREG, and EREG via ELISA using the following DuoSets from R&D, per the manufacturer’s instructions: CXCL-8 (Human IL-8/CXCL8 DuoSet ELISA, DY208); IL-6 (Human IL-6 DuoSet ELISA, DY206); AREG (Human Amphiregulin DuoSet ELISA, DY262); EREG (Human Epiregulin DuoSet ELISA, DY1195-05).

### 4.6. Proliferation Assay

The proliferation of an immortalized epithelial cell line in 2D culture treated with HEMF supernatants was determined with a colorimetric assay (MTT Cell Proliferation Assay Kit (Colorimetric), BioVision, AB211091-1000) in the presence and absence of the following neutralizing antibodies (R&D Systems) and concentrations: CXCL-8 (AB-208-NA, 5 µg/mL) and AREG (AB-262-NA, 5 µg/mL). We treated HEMFs with acidic bile salts as described in the manuscript; given the incompatibility between HEMF and epithelial cell culture media, after the last treatment with acidic bile salts, HEMFs were placed overnight in KSFM media. The supernatant was collected the following day and used to treat epithelial cells in 96-well plates (2000 cell/well) for 24 h in the presence/absence of neutralizing antibodies against IL-8 and AREG. An MTT assay, which measures cellular metabolic activity, was used to determine cell proliferation.

### 4.7. Statistics

All experiments were performed in triplicate and data are presented as means ± SE. Data from two test groups were analyzed using Student’s two-tailed type 2 *t*-test (Excel; Microsoft) or an ANOVA with Tukey’s post hoc test with GraphPad Prism 6.0 (GraphPad Software, La Jolla, CA, USA). *p*-value < 0.05 indicates statistical significance.

Materials, data, and associated protocols are available upon request. The datasets generated and/or analyzed during the current study have been deposited in NCBI’s Gene Expression Omnibus (Edgar et al., 2002) and are accessible through GEO Series accession number GSE197318.

## 5. Conclusions

The human esophageal stroma remains understudied. For the first time, we focused on the contribution of human esophageal myofibroblasts (HEMFs) and showed via an unbiased approach the response of HEMFs to injurious luminal insults encountered via the impaired epithelial barrier in GERD. In response to acidic bile salt, there was an activation of several inflammatory and proliferative pathways that involved paracrine mediators including HEMF-derived CXCL-8. These mediators can potentially have autocrine effects as well as paracrine effects. As such, cell–cell signaling, focusing on the effects of HEMFs on surrounding nonimmune stromal cells, immune cells, and adjacent epithelial cells in the esophagus warrant continued investigations.

## Data Availability

Materials, data, and associated protocols are available upon request. The datasets generated and/or analyzed during the current study have been deposited in NCBI’s Gene Expression Omnibus (Edgar et al., 2002) and are accessible through GEO Series accession number GSE197318.

## Supplementary Materials

The supporting information can be downloaded at: https://www.mdpi.com/article/10.3390/ijms231810371/s1.
