# Supplementary material for: Inflammatory and Proliferative Pathway Activation in Human Esophageal Myofibroblasts Treated with Acidic Bile Salts
Source: Int J Mol Sci. 2022 Sep 8;23(18):10371. doi: 10.3390/ijms231810371 (PMC9498994; doi:10.3390/ijms231810371)
Supplement: Supplementary file 1 [file ijms-23-10371-s001.zip › ijms-1820568-supplementary.pdf]

Table S1. Top differentially expressed genes with extracellular location ($p \leq 0.05$, fold change ≥1.2 or ≤ -1.2), organized by function (Cytokine, Growth factor, Enzymes, Peptidases, Kinases, Transporter, Other).

Gene Symbol	Gene name	Fold change	P-value
**Cytokine (n=13)**			
CXCL8	C-X-C motif chemokine ligand 8	53.229	0.00541
CXCL5	C-X-C motif chemokine ligand 5	12.520	0.0497
CCL5	C-C motif chemokine ligand 5	5.857	0.0349
CXCL6	C-X-C motif chemokine ligand 6	5.433	0.0305
CXCL3	C-X-C motif chemokine ligand 3	4.306	0.0348
IL33	interleukin 33	2.969	0.00672
LIF	LIF interleukin 6 family cytokine	1.475	0.015
WNT5A	Wnt family member 5A	1.427	0.0112
SPRED3	sprouty related EVH1 domain containing 3	1.393	0.0122
CLCF1	cardiotrophin like cytokine factor 1	1.317	0.00384
TNFSF18	TNF superfamily member 18	1.272	0.0181
CMTM3	CKLF like MARVEL transmembrane domain containing 3	-1.268	0.0322
TRIP6	thyroid hormone receptor interactor 6	-1.274	0.0194
**Growth factor (n=13)**			
AREG	amphiregulin	3.756	0.00113
FGF16	fibroblast growth factor 16	2.609	0.00811
ESM1	endothelial cell specific molecule 1	2.579	0.00683
EREG	epiregulin	2.465	0.0214
DKK1	dickkopf WNT signaling pathway inhibitor 1	1.758	0.00746
VEGFA	vascular endothelial growth factor A	1.586	0.0108

INHBA	inhibin subunit beta A	1.566	0.0442
BDNF	brain derived neurotrophic factor	1.42	0.0276
VEGFC	vascular endothelial growth factor C	1.24	0.0125
VEGFB	vascular endothelial growth factor B	-1.235	0.0479
LTBP4	latent transforming growth factor beta binding protein 4	-1.508	0.0454
BMP4	bone morphogenetic protein 4	-1.618	0.0306
JAG2	jagged canonical Notch ligand 2	-1.697	0.0394
**Enzymes (n=8)**			
CREG2	cellular repressor of E1A stimulated genes 2	1.854	0.0108
LIPG	lipase G, endothelial type	1.648	0.00552
NUDT6	nudix hydrolase 6	1.576	0.0487
CHI3L1	chitinase 3 like 1	-1.244	0.0407
TXNDC16	thioredoxin domain containing 16	-1.312	0.0287
CA11	carbonic anhydrase 11	-1.673	0.00186
CEL	carboxyl ester lipase	-2.253	0.0385
ART5	ADP-ribosyltransferase 5	-3.811	0.00434
**Peptidase (n=10)**			
MMP1	matrix metallopeptidase 1	3.698	0.0268
PLGLB1/PLGLB2	plasminogen like B2	2.602	0.0396
CTRB1	chymotrypsinogen B1	2.170	0.0344
PRSS3	serine protease 3	2.059	0.00765
PAPPA	pappalysin 1	1.710	0.0199
MMP3	matrix metallopeptidase 3	1.281	0.0108
MASP1	MBL associated serine protease 1	-1.232	0.00878
PRSS12	serine protease 12	-1.401	0.0028
MMP11	matrix metallopeptidase 11	-1.595	0.0443

PROC	protein C, inactivator of coagulation factors Va and VIIIa	-2.265	0.0388
**Kinases (n=2)**			
STC1	stanniocalcin 1	3.422	0.00237
CRIM1	cysteine rich transmembrane BMP regulator 1	-1.212	0.00319
**Transporter (n=3)**			
SEC23B	SEC23 homolog B, COPII coat complex component	1.431	0.0429
SLC39A8	solute carrier family 39 member 8	1.329	0.00258
APOD	apolipoprotein D	-1.484	0.0146
**Other (n=49)**			
CST2	cystatin SA	4.225	0.0159
DAW1	dynein assembly factor with WD repeats 1	3.387	0.0104
IGFBP1	insulin like growth factor binding protein 1	2.969	0.0283
CST1	cystatin SN	2.956	0.0122
ITIH1	inter-alpha-trypsin inhibitor heavy chain 1	2.508	0.0111
SERPIND1	serpin family D member 1	2.433	0.0181
TNFAIP6	TNF alpha induced protein 6	2.086	0.00584
PTHLH	parathyroid hormone like hormone	1.965	0.0374
TFPI2	tissue factor pathway inhibitor 2	1.738	0.0389
ANGPTL4	angiopoietin like 4	1.609	0.0318
MANF	mesencephalic astrocyte derived neurotrophic factor	1.595	0.00437
SERPINE1	serpin family E member 1	1.501	0.0322
UCN2	urocortin 2	1.49	0.000552
COL7A1	collagen type VII alpha 1 chain	1.441	0.0397

SEMA3A	semaphorin 3A	1.395	0.000671
CRYBG1	crystallin beta-gamma domain containing 1	1.372	0.0156
PTX3	pentraxin 3	1.327	0.0356
SERPINE2	serpin family E member 2	1.304	0.0459
SLIT2	slit guidance ligand 2	1.244	0.0384
GAL	galanin and GMAP prepropeptide	1.232	0.0217
LTBP1	latent transforming growth factor beta binding protein 1	1.228	0.00154
SMTN	smoothelin	1.21	0.0266
CCN1	cellular communication network factor 1	-1.203	0.00982
SEMA3E	semaphorin 3E	-1.204	0.0159
FRY	FRY microtubule binding protein	-1.225	0.00694
COL3A1	collagen type III alpha 1 chain	-1.231	0.0132
FAM210B	family with sequence similarity 210 member B	-1.233	0.0374
THBS1	thrombospondin 1	-1.269	0.0109
DMKN	dermokine	-1.271	0.0429
CDC42EP1	CDC42 effector protein 1	-1.285	0.0222
METTL25	methyltransferase like 25	-1.306	0.0497
PLEKHH3	pleckstrin homology, MyTH4 and FERM domain containing H3	-1.322	0.033
IGSF10	immunoglobulin superfamily member 10	-1.391	0.0368
SEMA3F	semaphorin 3F	-1.395	0.00368
VIT	vitrin	-1.417	0.021
ADM	adrenomedullin	-1.431	0.00838
COL8A2	collagen type VIII alpha 2 chain	-1.441	0.0323
CHAD	chondroadherin	-1.478	0.0236

SEMA3B	semaphorin 3B	-1.496	0.0213
SESN3	sestrin 3	-1.527	0.0297
COL14A1	collagen type XIV alpha 1 chain	-1.553	0.0314
MAMDC2	MAM domain containing 2	-1.586	0.00733
ECM2	extracellular matrix protein 2	-1.649	0.0474
RSPO2	R-spondin 2	-1.673	0.014
MATN2	matrilin 2	-1.712	0.0328
FAM184A	family with sequence similarity 184 member A	-1.816	0.0182
TAC3	tachykinin precursor 3	-1.86	0.0298
ERMN	ermin	-2.063	0.00204
IL34	interleukin 34	-3.407	0.00459

Table S2. Top differentially expressed genes (p value <= 0.05 and fold change ≥1.2 or ≤ -1.2) with a plasma membrane location, organized by function (Transporter, transmembrane receptor, phosphatase, peptidase, other, kinase, ion channel, growth factor, G-protein coupled receptor, enzyme, other).

Gene Symbol	Gene name	Fold change	P-value
**Transporter (n=34)**			
SLCO5A1	solute carrier organic anion transporter family member 5A1	4.647	0.00068
SLC19A3	solute carrier family 19 member 3	2.546	0.032
ANO3	anoctamin 3	2.262	0.0307
SLC6A15	solute carrier family 6 member 15	2.081	0.0109
SLC20A1	solute carrier family 20 member 1	1.63	0.0171
STX11	syntaxin 11	1.568	0.0228
SYN1	synapsin I	1.473	0.0426
SLC38A5	solute carrier family 38 member 5	1.43	0.0313
ANO7	anoctamin 7	1.382	0.00616

FLVCR2	FLVCR heme transporter 2	1.358	0.0102
ABCG2	ATP binding cassette subfamily G member 2 (Junior blood group)	1.331	0.0406
SLC7A2	solute carrier family 7 member 2	1.283	0.0305
SLC50A1	solute carrier family 50 member 1	1.253	0.0436
SLC7A5	solute carrier family 7 member 5	1.251	0.033
SLC16A3	solute carrier family 16 member 3	1.248	0.00537
SLC2A6	solute carrier family 2 member 6	1.238	0.0135
SLC12A6	solute carrier family 12 member 6	1.221	0.046
ANKH	ANKH inorganic pyrophosphate transport regulator	1.22	0.0408
ABCA3	ATP binding cassette subfamily A member 3	1.217	0.00337
GJD3	gap junction protein delta 3	-1.215	0.0415
SLC4A7	solute carrier family 4 member 7	-1.217	0.0444
SLC8B1	solute carrier family 8 member B1	-1.268	0.000651
ABCA5	ATP binding cassette subfamily A member 5	-1.283	0.00324
ATP8A2	ATPase phospholipid transporting 8A2	-1.312	0.0409
SLC43A1	solute carrier family 43 member 1	-1.342	0.0279
SLC2A12	solute carrier family 2 member 12	-1.427	0.0238
SYNGR1	synaptogyrin 1	-1.452	0.0249
SLC40A1	solute carrier family 40 member 1	-1.473	0.0182
SLC27A1	solute carrier family 27 member 1	-1.484	0.0474
SLC4A3	solute carrier family 4 member 3	-1.502	0.00314
SLC7A8	solute carrier family 7 member 8	-1.534	0.0272
SLC52A1	solute carrier family 52 member 1	-1.572	0.0415
ATP1A3	ATPase Na+/K+ transporting subunit alpha 3	-2.096	0.0436

SLC27A6	solute carrier family 27 member 6	-2.756	0.00201
**Transmembrane receptor (n=34)**			
HLA-DRA	major histocompatibility complex, class II, DR alpha	150.402	0.023
CD74	CD74 molecule	10.971	0.0222
IL7R	interleukin 7 receptor	1.941	0.0426
TREM1	triggering receptor expressed on myeloid cells 1	1.938	0.0293
HMMR	hyaluronan mediated motility receptor	1.873	0.0296
DNER	delta/notch like EGF repeat containing	1.758	0.0259
IL21R	interleukin 21 receptor	1.67	0.0265
ITGA2	integrin subunit alpha 2	1.645	0.0268
LRP8	LDL receptor related protein 8	1.561	0.0483
PLXNA4	plexin A4	1.555	0.0329
ULBP2	UL16 binding protein 2	1.431	0.029
CLDN4	claudin 4	1.421	0.00373
IL13RA2	interleukin 13 receptor subunit alpha 2	1.386	0.00785
ULBP1	UL16 binding protein 1	1.382	0.0313
OSMR	oncostatin M receptor	1.341	0.028
ITGA5	integrin subunit alpha 5	1.276	0.0132
IL15RA	interleukin 15 receptor subunit alpha	1.244	0.0384
IL6ST	interleukin 6 cytokine family signal transducer	1.219	0.0228
ITGA6	integrin subunit alpha 6	1.207	0.00992
IL4R	interleukin 4 receptor	1.202	0.0235
PTCH1	patched 1	-1.225	0.0273
GHR	growth hormone receptor	-1.28	0.0175
LIFR	LIF receptor subunit alpha	-1.29	0.0152

LRP3	LDL receptor related protein 3	-1.327	0.0482
PLXNB1	plexin B1	-1.348	0.0412
CD302	CD302 molecule	-1.357	0.0442
IL6R	interleukin 6 receptor	-1.399	0.0176
COLEC12	collectin subfamily member 12	-1.427	0.0149
TNFRSF10C	TNF receptor superfamily member 10c	-1.462	0.00937
HLA-DMA	major histocompatibility complex, class II, DM alpha	-1.483	0.0278
BCAM	basal cell adhesion molecule (Lutheran blood group)	-1.687	0.0424
C1QTNF5	C1q and TNF related 5	-1.737	0.0188
PLXNC1	plexin C1	-2.015	0.0134
SEMA6A	semaphorin 6A	-2.098	0.0354
**Phosphatase (n=3)**			
NT5E	5'-nucleotidase ecto	1.524	0.0131
TNS3	tensin 3	-1.287	0.0415
PTPRS	protein tyrosine phosphatase receptor type S	-1.299	0.00798
**Peptidase (n=3)**			
ANPEP	alanyl aminopeptidase, membrane	1.282	0.0296
DPP4	dipeptidyl peptidase 4	-1.277	0.00129
ACE	angiotensin I converting enzyme	-1.377	0.00806
**Kinase (n=9)**			
EPHB1	EPH receptor B1	1.925	0.0202
PODXL	podocalyxin like	1.548	0.00116
MPP2	membrane palmitoylated protein 2	1.213	0.0149
PDGFRB	platelet derived growth factor receptor beta	-1.24	0.0404

ACVR2B	activin A receptor type 2B	-1.254	0.0421
PTK7	protein tyrosine kinase 7 (inactive)	-1.262	0.0154
FGFR4	fibroblast growth factor receptor 4	-1.343	0.0031
EPHB3	EPH receptor B3	-1.587	0.0135
EFNB3	ephrin B3	-2.129	0.00807
**Ion channel (n=18)**			
KCNJ15	potassium inwardly rectifying channel subfamily J member 15	2.415	0.000276
CACNG6	calcium voltage-gated channel auxiliary subunit gamma 6	1.733	0.0066
KCNQ5	potassium voltage-gated channel subfamily Q member 5	1.525	0.047
TRPV3	transient receptor potential cation channel subfamily V member 3	1.385	0.00674
LRRC8C	leucine rich repeat containing 8 VRAC subunit C	1.248	0.00971
LRRC8E	leucine rich repeat containing 8 VRAC subunit E	-1.201	0.0068
CACNA1A	calcium voltage-gated channel subunit alpha1 A	-1.236	0.0474
CACNB3	calcium voltage-gated channel auxiliary subunit beta 3	-1.283	0.0424
ASIC1	acid sensing ion channel subunit 1	-1.327	0.00731
CACNA1C	calcium voltage-gated channel subunit alpha1 C	-1.422	0.00615
KCNS2	potassium voltage-gated channel modifier subfamily S member 2	-1.5	0.0164
KCNMB4	potassium calcium-activated channel subfamily M regulatory beta subunit 4	-1.508	0.0364
KCND1	potassium voltage-gated channel subfamily D member 1	-1.539	0.00946

GRIN3B	glutamate ionotropic receptor NMDA type subunit 3B	-1.656	0.0295
KCND3	potassium voltage-gated channel subfamily D member 3	-1.82	0.00416
FXYD1	FXYD domain containing ion transport regulator 1	-2.265	0.0249
GRIK4	glutamate ionotropic receptor kainate type subunit 4	-4.471	0.0129
KCNE1	potassium voltage-gated channel subfamily E regulatory subunit 1	-6.390	0.0109
**Growth factor (n=1)**			
NRG1	neuregulin 1	2.247	0.0306
**G-protein coupled receptor (n=26)**			
ADGRF4	adhesion G protein-coupled receptor F4	2.274	0.00157
HTR7	5-hydroxytryptamine receptor 7	1.853	0.000831
ADGRG1	adhesion G protein-coupled receptor G1	1.654	0.0333
ADGRL4	adhesion G protein-coupled receptor L4	1.612	0.00994
F2RL1	F2R like trypsin receptor 1	1.588	0.0125
PTGFR	prostaglandin F receptor	1.546	0.0339
GPR160	G protein-coupled receptor 160	1.545	0.0233
FZD8	frizzled class receptor 8	1.525	0.0326
GPR85	G protein-coupled receptor 85	1.513	0.039
GPR1	G protein-coupled receptor 1	1.484	0.0148
LTB4R2	leukotriene B4 receptor 2	1.228	0.0168
ADGRL2	adhesion G protein-coupled receptor L2	1.227	0.0296
AGTRAP	angiotensin II receptor associated protein	1.223	0.0125

LYPD1	LY6/PLAUR domain containing 1	1.206	0.0289
S1PR2	sphingosine-1-phosphate receptor 2	-1.214	0.0241
FZD7	frizzled class receptor 7	-1.26	0.0303
ADGRB2	adhesion G protein-coupled receptor B2	-1.271	0.0253
GPR162	G protein-coupled receptor 162	-1.31	0.0278
GPR20	G protein-coupled receptor 20	-1.449	0.0128
ADGRA2	adhesion G protein-coupled receptor A2	-1.6	0.0362
OPRL1	opioid related nociceptin receptor 1	-1.613	0.0118
GPR132	G protein-coupled receptor 132	-1.982	0.0269
PTH1R	parathyroid hormone 1 receptor	-2.022	0.0051
HTR2B	5-hydroxytryptamine receptor 2B	-2.283	0.029
GPR65	G protein-coupled receptor 65	-2.29	0.0314
OMG	oligodendrocyte myelin glycoprotein	-3.248	0.0396
**Enzymes (n=17)**			
GCNT3	glucosaminyl (N-acetyl) transferase 3, mucin type	1.613	0.0388
HAS2	hyaluronan synthase 2	1.6	0.00298
NCEH1	neutral cholesterol ester hydrolase 1	1.514	0.0108
C1GALT1	core 1 synthase, glycoprotein-N-acetylgalactosamine 3-beta-galactosyltransferase 1	1.418	0.022
GGT5	gamma-glutamyltransferase 5	1.306	0.029
CHMP1B	charged multivesicular body protein 1B	1.299	0.0169
GNG11	G protein subunit gamma 11	1.283	0.00377
ABHD17A	abhydrolase domain containing 17A, depalmitoylase	-1.254	0.0441
RHOQ	ras homolog family member Q	-1.256	0.0136

PLSCR4	phospholipid scramblase 4	-1.259	0.0422
RAP2B	RAP2B, member of RAS oncogene family	-1.281	0.0254
RAB40B	RAB40B, member RAS oncogene family	-1.298	0.0378
MAGI1	membrane associated guanylate kinase, WW and PDZ domain containing 1	-1.305	0.0163
GPNMB	glycoprotein nmb	-1.467	0.00498
CNTN4	contactin 4	-1.491	0.0176
GDPD5	glycerophosphodiester phosphodiesterase domain containing 5	-1.573	0.00934
GNGT2	G protein subunit gamma transducin 2	-1.806	0.0163
**Other (n=57)**			
ACE	angiotensin I converting enzyme	-1.377	0.00806
LHFPL5	LHFPL tetraspan subfamily member 5	3.922	0.0262
ESYT3	extended synaptotagmin 3	2.091	0.0217
NEFM	neurofilament medium	2.001	0.0189
MALL	mal, T cell differentiation protein like	1.808	0.0000148
CDCP1	CUB domain containing protein 1	1.783	0.0432
NTM	neurotrimin	1.723	0.00372
PLS1	plastin 1	1.675	0.0376
TPBG	trophoblast glycoprotein	1.491	0.011
TM4SF1	transmembrane 4 L six family member 1	1.474	0.00255
ESAM	endothelial cell adhesion molecule	1.38	0.0142
DCBLD2	discoidin, CUB and LCCL domain containing 2	1.309	0.00826
C12orf66	KICSTOR subunit 2	1.285	0.041

CD82	CD82 molecule	1.239	0.0313
XKR8	XK related 8	1.234	0.0213
ITPRID2	ITPR interacting domain containing 2	1.231	0.0255
ARRDC3	arrestin domain containing 3	1.225	0.0426
CD55	CD55 molecule (Cromer blood group)	1.209	0.00226
ROBO4	roundabout guidance receptor 4	1.208	0.0202
ASAP1	ArfGAP with SH3 domain, ankyrin repeat and PH domain 1	-1.201	0.0206
CLIP3	CAP-Gly domain containing linker protein 3	-1.204	0.0128
RECK	reversion inducing cysteine rich protein with kazal motifs	-1.213	0.00786
SDC2	syndecan 2	-1.215	0.0251
EVL	Enah/Vasp-like	-1.22	0.0332
TLCD2	TLC domain containing 2	-1.221	0.00909
DTNB	dystrobrevin beta	-1.226	0.0297
NTNG2	netrin G2	-1.236	0.045
FARP1	FERM, ARH/RhoGEF and pleckstrin domain protein 1	-1.253	0.0463
NISCH	nischarin	-1.254	0.0099
TSPAN6	tetraspanin 6	-1.255	0.00203
CCDC8	coiled-coil domain containing 8	-1.257	0.0481
KLHL24	kelch like family member 24	-1.258	0.00691
KAZN	kazrin, periplakin interacting protein	-1.282	0.0006
MDGA1	MAM domain containing glycosylphosphatidylinositol anchor 1	-1.29	0.0288
CCDC120	coiled-coil domain containing 120	-1.323	0.0106
PARD3B	par-3 family cell polarity regulator beta	-1.327	0.00425
AMOT	angiomotin	-1.335	0.0208

AMOTL2	angiomotin like 2	-1.338	0.00212
INSYN1	inhibitory synaptic factor 1	-1.37	0.0485
ANKRD13B	ankyrin repeat domain 13B	-1.419	0.0164
MELTF	melanotransferrin	-1.476	0.0352
PCDHB5	protocadherin beta 5	-1.502	0.0263
PALM	paralemmin	-1.513	0.0348
DLGAP1	DLG associated protein 1	-1.521	0.00713
EFNB1	ephrin B1	-1.542	0.0363
PLXDC1	plexin domain containing 1	-1.569	0.0381
ANK1	ankyrin 1	-1.594	0.011
VSIR	V-set immunoregulatory receptor	-1.613	0.027
SEMA4G	semaphorin 4G	-1.65	0.00403
GAS1	growth arrest specific 1	-1.699	0.021
CLDN11	claudin 11	-1.728	0.0241
EEPD1	endonuclease/exonuclease/phosphatase family domain containing 1	-1.747	0.0437
RIMS4	regulating synaptic membrane exocytosis 4	-1.778	0.00755
SEMA4A	semaphorin 4A	-1.974	0.0293
AKAP5	A-kinase anchoring protein 5	-2.087	0.0304
KNDC1	kinase non-catalytic C-lobe domain containing 1	-2.211	0.000296
TSPAN7	tetraspanin 7	-2.353	0.0272
LRRN2	leucine rich repeat neuronal 2	-2.808	0.00423

Table S3. Molecules associated with the Agranulocyte Adhesion and Diapedesis pathway and Neuregulin Signaling canonical pathways. Enriched pathways are based on p-value < 0.05.

Agranulocyte Adhesion and Diapedesis					
**Gene symbol**	**Gene Name**	**Fold change**	**p-value**	**Location**	**type**
CXCL8	C-X-C motif chemokine ligand 8	53.229	0.00541	Extracellular Space	cytokine
CXCL5	C-X-C motif chemokine ligand 5	12.52	0.0497	Extracellular Space	cytokine
CCL5	C-C motif chemokine ligand 5	5.857	0.0349	Extracellular Space	cytokine
CXCL6	C-X-C motif chemokine ligand 6	5.433	0.0305	Extracellular Space	cytokine
CXCL3	C-X-C motif chemokine ligand 3	4.306	0.0348	Extracellular Space	cytokine
MMP1	matrix metallopeptidase 1	3.698	0.0268	Extracellular Space	peptidase
IL33	interleukin 33	2.969	0.00672	Extracellular Space	cytokine
ITGA2	integrin subunit alpha 2	1.645	0.0268	Plasma Membrane	transmembrane receptor
PODXL	podocalyxin like	1.548	0.00116	Plasma Membrane	kinase
CLDN4	claudin 4	1.421	0.00373	Plasma Membrane	transmembrane receptor
MMP3	matrix metallopeptidase 3	1.281	0.0108	Extracellular Space	peptidase
ITGA5	integrin subunit alpha 5	1.276	0.0132	Plasma Membrane	transmembrane receptor
ITGA6	integrin subunit alpha 6	1.207	0.00992	Plasma Membrane	transmembrane receptor
MYH10	myosin heavy chain 10	-1.274	0.00228	Cytoplasm	enzyme
ACTA2	actin alpha 2, smooth muscle	-1.31	0.0208	Cytoplasm	other
MMP11	matrix metallopeptidase 11	-1.595	0.0443	Extracellular Space	peptidase
MYH11	myosin heavy chain 11	-1.61	0.0265	Cytoplasm	other
CLDN11	claudin 11	-1.728	0.0241	Plasma Membrane	other

Neuregulin					
**Gene symbol**	**Gene Name**	**Fold change**	**p-value**	**Location**	**type**

AREG	amphiregulin	3.756	0.00113	Extracellular Space	growth factor
CDK5	cyclin dependent kinase 5	1.247	0.0192	Nucleus	kinase
CDKN1B	cyclin dependent kinase inhibitor 1B	-1.283	0.00159	Nucleus	kinase
EREG	epiregulin	2.465	0.0214	Extracellular Space	growth factor
ERRFI1	ERBB receptor feedback inhibitor 1	-1.269	0.0171	Cytoplasm	other
HSP90AA1	heat shock protein 90 alpha family class A member 1	1.227	0.028	Cytoplasm	enzyme
HSP90AB1	heat shock protein 90 alpha family class B member 1	1.205	0.0108	Cytoplasm	enzyme
HSP90B1	heat shock protein 90 beta family member 1	1.274	0.0148	Cytoplasm	other
ITGA2	integrin subunit alpha 2	1.645	0.0268	Plasma Membrane	transmembrane receptor
ITGA5	integrin subunit alpha 5	1.276	0.0132	Plasma Membrane	transmembrane receptor
ITGA6	integrin subunit alpha 6	1.207	0.00992	Plasma Membrane	transmembrane receptor
NRG1	neuregulin 1	2.247	0.0306	Plasma Membrane	growth factor
PLCG2	phospholipase C gamma 2	-1.948	0.000912	Cytoplasm	enzyme
RAP2B	RAP2B, member of RAS oncogene family	-1.281	0.0254	Plasma Membrane	enzyme
TMEFF2	transmembrane protein with EGF like and two follistatin like domains 2	1.636	0.0245	Cytoplasm	other

Table S4. Top Upstream regulators by IPA with p-value of overlap < 0.05. when available, predicted activation state and z-score are shown where a z score ≥ 2 is predicted as an activated upstream regulator whereas z score ≤ -2 is inhibited regulator.

**Upstream Regulator**	**Expr Fold Change**	**Molecule Type**	**Predicted Activation State**	**Activation z-score**	**p-value of overlap**
ERBB2	-1.151	kinase	Activated	5.716	3.28E-16
CG		complex	Activated	4.938	9.67E-15
TNF		cytokine	Activated	5.263	3.13E-13
TGFB1	1.195	growth factor		1.819	3.41E-13
IGF1	1.164	growth factor	Activated	3.407	3.45E-13
HGF	1.307	growth factor	Activated	5.242	5.78E-13
Vegf		group	Activated	4.179	6.56E-13
EGFR	1.011	kinase	Activated	2.726	2.24E-11
EGF	-1.275	growth factor	Activated	3.73	9.13E-11

Pkc(s)		group		0.945	1.1E-10
TP53	-1.099	transcription regulator	Inhibited	-4.57	2.76E-10
F2	2.858	peptidase	Activated	3.036	3.52E-10
HRAS	1.013	enzyme	Activated	3.166	7.38E-10
IL1B	7.277	cytokine	Activated	5.445	1.72E-09
AHR	-1.002	ligand-dependent nuclear receptor		0.403	5.75E-09
CDKN1A	-1.024	kinase	Inhibited	-4.237	6.03E-09
MAP2K1	1.104	kinase	Activated	2.059	1.43E-08
ESR2		ligand-dependent nuclear receptor	Activated	3.597	1.85E-08
HDAC4	-1.082	transcription regulator		0.231	2.06E-08
FGF2	1.139	growth factor	Activated	2.425	5.87E-08
PTGER2	-1.27	G-protein coupled receptor	Activated	2.892	6.05E-08
IL1		group	Activated	3.917	8.15E-08
MRTFB	1.008	transcription regulator	Inhibited	-2.199	8.48E-08
GNA14	1.073	enzyme			9.29E-08
NFkB (complex)		complex	Activated	3.959	0.000000104
RAF1	-1.032	kinase	Activated	3.083	0.000000106
TCF3	-1.159	transcription regulator		-1.791	0.000000108
ZBTB17	1.075	transcription regulator			0.000000149
SMARCA 4	1.018	transcription regulator		1.771	0.000000186
PDGF BB		complex	Activated	3.008	0.0000002
ADRB		group		1.675	0.000000263
E2F4	-1.004	transcription regulator			0.000000309
IL1A	4.178	cytokine	Activated	3.262	0.000000545
NFKBIA	1.49	transcription regulator		1.201	0.000000599
CDKN2A	-1.298	transcription regulator	Inhibited	-3.042	0.000000619
Mek		group	Activated	3.77	0.000000643
RRP1B	1.001	transcription regulator			0.000000656
FOS	5.333	transcription regulator	Activated	2.653	0.000000722
IL6	1.61	cytokine	Activated	2.646	0.000000754
IL17A		cytokine	Activated	2.674	0.000000957

E2F1	1.436	transcription regulator		1.573	0.00000098
CREM	1.122	transcription regulator		-0.304	0.00000106
NRG1	2.247	growth factor		1.371	0.00000113
CCN1	-1.203	other	Activated	2.269	0.00000113
TREM1	1.938	transmembrane receptor	Activated	3.005	0.00000126
IGF2	-1.013	growth factor		0.938	0.00000129
NUPR1	-1.198	transcription regulator		-0.128	0.00000133
WNT1		cytokine		-0.461	0.00000151
P38 MAPK		group		1.833	0.00000156
let-7		microRNA	Inhibited	-2.843	0.0000016
SP1	-1.054	transcription regulator	Activated	2.759	0.00000161
AR	-1.136	ligand-dependent nuclear receptor		1.652	0.00000178
RB1	1.022	transcription regulator		-1.767	0.00000193
ERK1/2		group	Activated	3.393	0.00000251
GNAQ	-1.012	enzyme		0.651	0.0000029
OSM		cytokine	Activated	3.243	0.00000359
CSF2		cytokine	Activated	4.895	0.00000435
ERK		group		1.802	0.00000457
YY1	-1.081	transcription regulator		0.888	0.0000049
RABL6	-1.066	other	Activated	3.973	0.0000054
CREB1	-1.012	transcription regulator	Activated	2.042	0.0000063
SFTPA1		transporter	Inhibited	-2.021	0.00000675
TEAD4	-1.013	transcription regulator		-0.278	0.00000713
FOXO3	-1.173	transcription regulator		-0.899	0.0000074
Rb		group	Inhibited	-3.606	0.00000847
MAPK8	-1.094	kinase		0.195	0.0000085
CKS1B	1.477	kinase	Activated	2.204	0.0000091
PLA2G5		enzyme		0.598	0.0000091
ERBB3	-1.372	kinase		0.974	0.00000922
NOTCH3	-1.06	transcription regulator		-0.867	0.0000108
BSG	-1.027	transporter	Activated	3.214	0.0000112
BMP15		growth factor		1.468	0.0000124
PIK3R1	-1.368	kinase	Activated	2.58	0.0000125

IFNG		cytokine		1.763	0.0000128
TCF4	-1.117	transcription regulator		-0.224	0.0000155
DICER1	-1.038	enzyme		-0.309	0.0000161
EDN1	-1.133	cytokine	Activated	2.298	0.0000164
APP	-1.092	other		1.9	0.0000175
KRAS	-1.171	enzyme	Activated	3.273	0.0000193
Pdgf (complex)		complex	Activated	2.542	0.0000199
INHA		growth factor		-0.548	0.0000243
ADRA1A		G-protein coupled receptor			0.0000244
MRTFA	-1.039	transcription regulator		-1.815	0.0000252
CDK2	1.206	kinase		0.378	0.00665

Table S5. Target molecules in dataset regulated by ERBB2 upstream regulator; ERBB2 predicted to be activated with z-score 5.716, overlap p-value 3.28E-16; 69 of 114 genes have measurement direction consistent with activation of ERBB2.

Genes in dataset	Prediction (based on measurement direction)	Expr Fold Change	Findings
CXCL8	Activated	53.229	Upregulates
CCL5	Activated	5.857	Upregulates
CXCL3	Activated	4.306	Upregulates
AREG	Activated	3.756	Upregulates
MMP1	Activated	3.698	Upregulates
PTGS2	Activated	2.557	Upregulates
EREG	Activated	2.465	Upregulates
NRG1	Affected	2.247	Regulates
SLC6A15	Activated	2.081	Upregulates
PRSS3	Activated	2.059	Upregulates
TREM1	Activated	1.938	Upregulates
NDC80	Activated	1.817	Upregulates
CDCP1	Activated	1.783	Upregulates
NEK2	Affected	1.757	Regulates
CDC6	Activated	1.727	Upregulates
GDPD3	Affected	1.689	Regulates
ITGA2	Inhibited	1.645	Downregulates
ANGPTL4	Activated	1.609	Upregulates
HAS2	Inhibited	1.6	Downregulates
TOP2A	Activated	1.599	Upregulates
VEGFA	Activated	1.586	Upregulates
CCNA2	Affected	1.58	Regulates
ORC6	Activated	1.578	Upregulates

UBE2C	Activated	1.577	Upregulates
CDCA8	Activated	1.577	Upregulates
SPAG5	Activated	1.576	Upregulates
ORC1	Activated	1.56	Upregulates
PODXL	Activated	1.548	Upregulates
MAPK13	Activated	1.539	Upregulates
NT5E	Activated	1.524	Upregulates
CDC7	Activated	1.521	Upregulates
GALNT18	Inhibited	1.503	Downregulates
SERPINE1	Affected	1.501	Regulates
BHLHE40	Activated	1.495	Upregulates
PRC1	Affected	1.494	Regulates
CKS1B	Affected	1.477	Regulates
TNFAIP3	Activated	1.462	Upregulates
GINS3	Activated	1.46	Upregulates
PTGS1	Inhibited	1.457	Downregulates
PDIA4	Affected	1.449	Regulates
COL7A1	Activated	1.441	Upregulates
PHLDA2	Activated	1.433	Upregulates
FEN1	Activated	1.432	Upregulates
ZWINT	Activated	1.428	Upregulates
WNT5A	Affected	1.427	Regulates
CLDN4	Activated	1.421	Upregulates
RFC4	Activated	1.419	Upregulates
CCNB1	Activated	1.406	Upregulates
CDC20	Activated	1.4	Upregulates
LXN	Affected	1.394	Regulates
RAD51AP1	Affected	1.391	Regulates
E2F7	Activated	1.391	Upregulates
ABHD5	Affected	1.387	Regulates
CENPE	Affected	1.386	Regulates
LDHA	Activated	1.384	Upregulates
WWC1	Affected	1.377	Regulates
CRYBG1	Affected	1.372	Regulates
PORCN	Activated	1.367	Upregulates
POLD3	Activated	1.347	Upregulates
NPC1	Inhibited	1.339	Downregulates
HIF1A	Activated	1.335	Upregulates
ABCG2	Affected	1.331	Regulates
POLR2J2/POLR2J3	Inhibited	1.324	Downregulates
DDX10	Affected	1.313	Regulates
GGT5	Activated	1.306	Upregulates
POLD4	Inhibited	1.287	Downregulates
MMP3	Activated	1.281	Upregulates
ITGA5	Activated	1.276	Upregulates

JUNB	Activated	1.271	Upregulates
PAICS	Activated	1.262	Upregulates
RFC2	Activated	1.257	Upregulates
SLC7A5	Inhibited	1.251	Downregulates
VEGFC	Activated	1.24	Upregulates
CD82	Affected	1.239	Regulates
MCM4	Activated	1.235	Upregulates
MCM3	Activated	1.226	Upregulates
IL6ST	Activated	1.219	Upregulates
POLR3K	Activated	1.216	Upregulates
BARD1	Affected	1.214	Regulates
RRM1	Activated	1.214	Upregulates
RPA2	Activated	1.212	Upregulates
SMTN	Activated	1.21	Upregulates
PER1	Activated	1.21	Upregulates
ITGA6	Activated	1.207	Upregulates
CLU	Activated	-1.204	Downregulates
JUN	Inhibited	-1.211	Upregulates
RECK	Activated	-1.213	Downregulates
SLC4A7	Inhibited	-1.217	Upregulates
BCKDHB	Affected	-1.22	Regulates
GXYLT2	Activated	-1.225	Downregulates
CHCHD10	Affected	-1.228	Regulates
COL3A1	Affected	-1.231	Regulates
SHROOM3	Affected	-1.258	Regulates
HSPB1	Inhibited	-1.259	Upregulates
THBS1	Activated	-1.269	Downregulates
GHR	Activated	-1.28	Downregulates
CDKN1B	Activated	-1.283	Downregulates
PTPRS	Activated	-1.299	Downregulates
MITF	Activated	-1.301	Downregulates
ACTA2	Inhibited	-1.31	Upregulates
SRPX	Affected	-1.311	Regulates
CCDC80	Affected	-1.312	Regulates
ST6GAL1	Activated	-1.322	Downregulates
FOXO1	Affected	-1.383	Regulates
IL6R	Inhibited	-1.399	Upregulates
ZFP36L2	Inhibited	-1.429	Upregulates
TPD52L1	Affected	-1.502	Regulates
RRAD	Activated	-1.579	Downregulates
MMP11	Affected	-1.595	Regulates
CDC42BPG	Inhibited	-1.63	Upregulates
BCAM	Activated	-1.687	Downregulates
JAG2	Inhibited	-1.697	Upregulates
EFNB3	Activated	-2.129	Downregulates
CBFA2T3	Affected	-2.489	Regulates
